# Association of polycystic ovary syndrome with metabolic syndrome and its components in adolescents: a systematic review and meta-analysis

**DOI:** 10.3389/fmed.2026.1736558

**Published:** 2026-03-26

**Authors:** Yuhui Tu, Yafei Chen, Jianwei Zhang, Jiaping Bao, Jianbo Lou

**Affiliations:** 1 Gynecology Department, Shaoxing Maternity and Child Health Care Hospital, Shaoxing, Zhejiang, China; 2 Pediatric Ward, Shaoxing Maternity and Child Health Care Hospital, Shaoxing, Zhejiang, China

**Keywords:** adolescents, meta-analysis, metabolic syndrome, polycystic ovary syndrome, systematic review

## Abstract

**Background:**

Polycystic ovary syndrome (PCOS) is a prevalent endocrine and metabolic disorder among adolescent girls, while metabolic syndrome (MetS) is a major precursor to cardiovascular disease and type 2 diabetes. The strength of the association between PCOS and MetS, as well as between PCOS and its core components, in adolescents remains unclear. This study aimed to conduct a systematic review and meta-analysis to clarify the risk of MetS and its individual metabolic abnormalities in adolescents with PCOS.

**Methods:**

We systematically searched PubMed, Embase, the Cochrane Library, and the Web of Science databases for observational studies published up to September 2025 that included adolescent girls aged 10–20 years, with and without PCOS. For categorical variables, odds ratios (ORs) with 95% confidence intervals (CIs) were calculated, while weighted mean differences (WMDs) with 95% CIs were used for continuous variables. All meta-analyses were performed using a random-effects model.

**Results:**

The final meta-analysis included 13 studies (5 cross-sectional, 6 case–control, and 2 cohort studies) comprising 1,789 participants (1,005 with PCOS and 784 controls). Pooled results indicated a significantly higher risk of MetS in adolescents with PCOS than in controls (OR: 2.61, 95% CI: 1.83–3.74, *p* < 0.001). Furthermore, the PCOS group exhibited significantly higher values for specific MetS components, including waist circumference (WMD: 3.23 cm, 95% CI: 0.91–5.55, *p* = 0.006), systolic blood pressure (WMD: 3.80 mmHg, 95% CI: 0.59–7.00, *p* = 0.020), and triglycerides (WMD: 5.76 mg/dL, 95% CI: 1.05–10.46, *p* = 0.017). In contrast, no statistically significant differences were observed in diastolic blood pressure, high-density lipoprotein, or fasting blood glucose levels.

**Conclusion:**

Adolescent PCOS is significantly associated with an elevated risk of MetS, with abnormalities primarily clustered in abdominal obesity, systolic blood pressure, and triglyceride levels. Integrating these three key metrics into routine metabolic screening for adolescents with PCOS is clinically essential. Prioritizing lifestyle interventions to address these risk factors is critical for mitigating long-term cardiometabolic complications.

**Systematic review registration:**

The study was registrated in International Platform of Registered Systematic Review and Meta-analysis Protocols (Registration number: INPLASY2025100048).

## Introduction

Adolescence represents a critical period for the maturation of the female reproductive endocrine system and the establishment of metabolic homeostasis. Polycystic ovary syndrome (PCOS), the most common endocrine and metabolic disorder in this age group, has a global prevalence of 3–8% (1). Its core pathological features include hyperandrogenism, ovulatory dysfunction, and polycystic ovarian morphology (2, 3). A key pathophysiological mechanism is decreased insulin sensitivity, which is closely associated with the development of metabolic disturbances (4).

Metabolic syndrome (MetS) is defined as a cluster of metabolic abnormalities centered around abdominal obesity, fasting hyperglycemia, dyslipidemia—characterized by elevated triglycerides and reduced high-density lipoprotein cholesterol [HDL-C], and hypertension (5). Clinically, MetS substantially increases the long-term risk of type 2 diabetes mellitus (T2DM) and cardiovascular disease (CVD) (6, 7). Although its prevalence among adolescents (approximately 2–5%) is lower than that in adults, its emergence during this developmental stage raises particular concern. Adverse metabolic profiles during adolescence may persist into adulthood due to a “metabolic memory” effect, thereby increasing the future risk of T2DM and CVD (8, 9). Therefore, identifying at-risk adolescents and clarifying the factors associated with MetS are essential for interrupting the trajectory of metabolic disease.

Growing observational evidence suggests that PCOS may be an important risk factor for MetS in adolescents (10). The unique physiological context of adolescence—marked by dynamic changes in insulin resistance, body composition, and hormonal levels—may shape a distinct relationship between PCOS and MetS that differs from that observed in adults (11). Current clinical management of adolescents with PCOS often prioritizes reproductive aspects, while screening and interventions for metabolic risks lack sufficient evidence-based guidance (12). There is a clear need to systematically integrate existing evidence to quantify the association between adolescent PCOS and MetS, including its specific components. Such efforts will provide the foundation for developing targeted metabolic screening strategies and early intervention protocols. Therefore, this study conducts a systematic review and meta-analysis to investigate the association between PCOS and MetS in adolescents, aiming to clarify the strength of this relationship and its potential influencing factors.

## Methods

### Data sources, search strategy, and selection criteria

This systematic review and meta-analysis was conducted in accordance with the PRISMA statement (13). The study protocol was prospectively registered on the International Platform of Registered Systematic Review and Meta-analysis Protocols (Registration number: INPLASY2025100048) to ensure transparency and traceability and to minimize the risk of selective reporting bias.

A comprehensive literature search was conducted using PubMed, Embase, the Cochrane Library, and Web of Science, including records from database inception until September 2025. The search strategy incorporated both controlled vocabulary (e.g., MeSH and Emtree) and free-text terms. Key search terms in the English language included “adolescent,” “teenager,” “youth*,” “polycystic ovary syndrome”, “PCOS”, “metabolic syndrome”, “MetS”, “insulin resistance”, and “abdominal obesity.” Complete, database-specific search strategies are provided in Supplementary File 1. To ensure comprehensive coverage, the reference lists of all identified primary studies and relevant reviews were manually screened for additional eligible records.

Two reviewers independently performed literature screening and study selection following a predefined protocol. Discrepancies were resolved through team discussion until consensus was reached. Studies were included if they met the following criteria: (1) Population: adolescent girls aged 10–20 years, with those in the PCOS group diagnosed according to internationally recognized criteria such as the Rotterdam Criteria, NIH criteria, ESHRE/ASRM guidelines, the 2023 International Evidence-based Guideline, or AE-PCOS Society Criteria for Adolescents; (2) Study design: published observational studies, including cross-sectional, case–control, or cohort designs; and (3) Outcomes: the primary outcome was the incidence of MetS, while secondary outcomes included incidence or association measures for individual MetS components. Studies were excluded if they included adults (≥20 years) or male patients without providing extractable data specific to the adolescent female subgroup; applied unclear or non-international diagnostic criteria for PCOS; did not use adolescent-specific criteria for diagnosing MetS; or were part of reviews, case reports, case series, animal studies, or *in vitro* investigations.

### Data collection and quality assessment

A standardized data extraction form was developed, and two researchers independently extracted the following information: first author, year of publication, country/region, study design, sample size, mean age, PCOS definition, MetS definition, incidence of MetS in the PCOS and control groups, and study outcomes. The quality of included studies was assessed using the Newcastle–Ottawa Scale (NOS) across three domains: “selection of study subjects” (four items), “comparability between groups” (two items), and “outcome/exposure measurement” (three items) (14). Any discrepancies in data extraction or quality assessment were resolved by a third researcher through a full-text review.

### Statistical analysis

Given that the included studies were primarily cross-sectional and case–control in design, odds ratios (ORs) with 95% confidence intervals (CIs) were used to express the association between PCOS and MetS. Weighted mean differences (WMDs) with 95% CIs were used to compare continuous variables between groups. All meta-analyses were performed using a random-effects model to account for potential heterogeneity among studies (15, 16). Heterogeneity was assessed using the *Q*-test and *I*^2^ statistic, with *I*^2^ ≥ 50% or a Q-test *p*-value of ≤0.10 indicating significant heterogeneity (17, 18). Sensitivity analysis was conducted sequentially by excluding each study and re-pooling the effect sizes to evaluate the stability of the results (19). Subgroup analyses were performed based on study design, geographical region, PCOS diagnostic criteria, MetS diagnostic criteria, and study quality. Differences in association strength across subgroups were tested using an interaction t-test, which assumes a normal distribution of the data (20). Publication bias was visually assessed using funnel plots and statistically evaluated using Egger’s linear regression test and Begg’s rank correlation test. If publication bias was detected, the trim-and-fill method was applied to adjust the effect size and evaluate its impact on the results (21–23). All statistical tests were two-sided, with a significance level of *α* = 0.05. Analyses were performed using Stata 18.0 (StataCorp, College Station, TX, USA).

## Results

### Literature search

**Figure 1:**
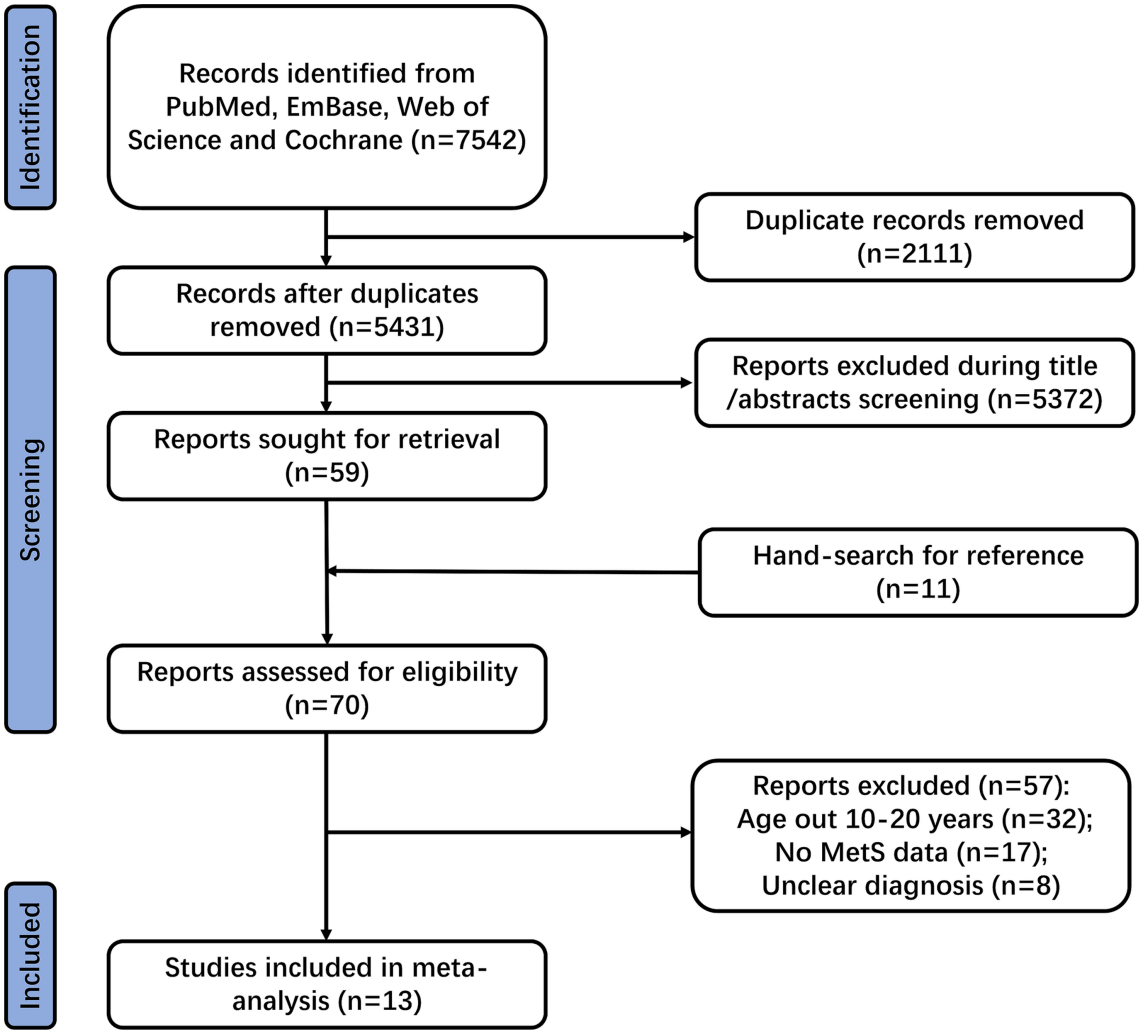
PRISMA 2020 flow diagram for the systematic review.

A total of 7,542 records were identified through electronic database searches. After removing duplicates, 5,431 articles remained. Initial screening of titles and abstracts excluded 5,372 articles due to mismatched study populations, irrelevant outcomes, or ineligible study types, leaving 59 articles for full-text review. After full-text assessment, 46 studies were excluded for the following reasons: failure to meet the age criteria (*n* = 21), absence of MetS outcome data (*n* = 17), and unclear diagnostic criteria (*n* = 8). Manual screening of their reference lists identified 11 additional potentially eligible studies, all of which were subsequently excluded as they included women over 20 years of age. Ultimately, 13 observational studies that met the eligibility criteria were included (24–36). The study selection process is illustrated in Figure 1.

### Study characteristics

The basic characteristics of the included studies are summarized in Table 1. The 13 eligible publications, spanning from 2008 to 2025, consisted of 5 cross-sectional studies, 6 case–control studies, and 2 cohort studies. The total sample size was 1,789 participants, including 1,005 individuals in the PCOS group and 784 in the control group. Regarding PCOS diagnostic criteria, 8 studies applied the Rotterdam or NIH criteria, while the remaining 5 studies used the ESHRE/ASRM criteria (3 studies), the AES-2006 criteria (1 study), or the 2023 International Evidence-based Guideline (1 study). All studies used adolescent-specific criteria for diagnosing MetS, with 8 of them using the International Diabetes Federation criteria. Study quality was assessed using the NOS, with scores of 7–9 indicating high quality and 5–6 indicating fair quality. Among the included studies, 5 were rated as high quality and 8 as fair quality.

**Table 1:** Baseline characteristics of the identified studies and the involved patients.

Study	Study design	Country	Sample size (PCOS/control)	Age (years)	PCOS definition	MetS definition	No of MetS (PCOS/control)	NOS
Rossi 2008 (24)	Cross-sectional	USA	74 (43/31)	15.6/14.8	Rotterdam	IDF	11/6	8
Huang 2010 (25)	Cross-sectional	China	168 (128/40)	18.0/19.0	Rotterdam/NIH	IDF	6/1	8
Hart 2011 (26)	Prospective cohort	Australia	204 (61/143)	15.1/15.1	Rotterdam	IDF	3/6	8
Vrbikova 2011 (27)	Case–control	Czech Republic	91 (43/48)	16.8/17.5	ESHRE/ASRM	IDF	5/1	5
Bhattacharya 2011 (28)	Cross-sectional	India	96 (51/45)	17.1/16.7	AES 2006 criteria	2009 ‘joint interim criteria’	31/12	7
Rahmanpour 2012 (29)	Case–control	Iran	101 (30/71)	17.7/17.7	NIH	IDF	10/8	6
Nandalike 2012 (30)	Case–control	USA	56 (28/28)	16.8/17.1	Rotterdam	Weiss criteria	10/4	5
Panidis 2013 (31)	Case–control	Greece	342 (332/10)	18.1/18.3	Rotterdam	IDF	100/0	5
Aydin 2015 (32)	Cross-sectional	Türkiye	222 (63/159)	15.7/16.4	ESHRE/ASRM	Modified Cook criteria	5/1	6
Han 2015 (33)	Case–control	The Republic of Korea	89 (49/40)	17.1/16.9	NIH	NCEP ATP III criteria	2/1	7
Oztas 2016 (34)	Case–control	Türkiye	172 (89/83)	18.4/18.8	ESHRE/ASRM	IDF	9/5	5
Keskin 2024 (35)	Cross-sectional	Türkiye	45 (19/26)	15.9/15.1	2023 IEG	Modified NCEP ATP III criteria	8/9	6
Kara 2025 (36)	Prospective cohort	Türkiye	132 (69/63)	15.7/15.7	Rotterdam	IDF	20/9	5

### MetS risk in PCOS

**Figure 2:**
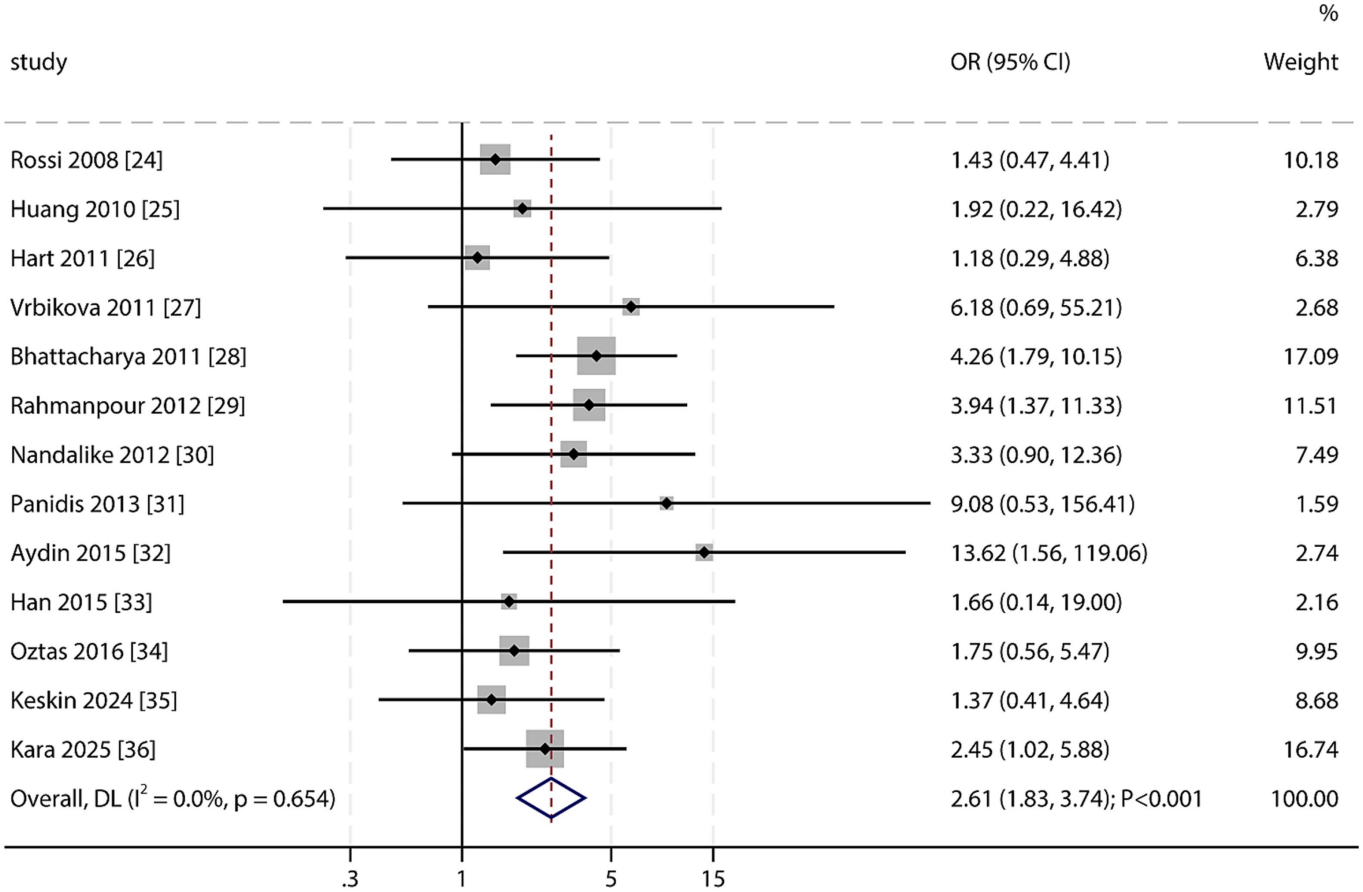
Forest plot of the association between PCOS and the risk of MetS. Weights are from random-effects model; continuity correction applied to studies with zero cells.

All 13 included studies provided effect size data for the association between PCOS and MetS. The pooled results demonstrated a significantly higher prevalence of MetS in adolescents with PCOS than in non-PCOS controls (OR: 2.61; 95% CI: 1.83–3.74; *p* < 0.001; Figure 2), with no significant heterogeneity observed across studies (*I*^2^ = 0.0%; *p* = 0.654). Sensitivity analysis confirmed that the pooled result was robust and not substantially affected by omitting any single study (Supplementary File 2). Subgroup analyses indicated a significantly increased risk of MetS in PCOS patients across the majority of subgroups, except for prospective cohort studies, in which the association was not statistically significant (Table 2). No significant publication bias was detected for the association between PCOS and MetS (Egger’s test *p* = 0.099; Begg’s test *p* = 0.945; Supplementary File 3).

**Table 2:** Subgroup analysis of the association between PCOS and the risk of MetS.

Factors	Subgroups	No of studies	OR and 95%CI	*p*-value	*I*^2^ (%)	*Q* statistic	Interaction test
Study design	Cross-sectional	5	2.59 (1.28–5.27)	0.008	29.9	0.222	0.677
	Case–control	6	3.09 (1.69–5.64)	<0.001	0.0	0.794	
	Prospective cohort	2	2.00 (0.95–4.22)	0.068	0.0	0.391	
Country	Eastern	4	3.65 (1.97–6.78)	<0.001	0.0	0.827	0.205
	Western	9	2.21 (1.42–3.43)	<0.001	0.0	0.525	
PCOS definition	Rotterdam/NIH	8	2.38 (1.49–3.79)	<0.001	0.0	0.798	0.530
	Other	5	3.03 (1.53–6.02)	0.002	25.5	0.251	
MetS definition	IDF	8	2.29 (1.45–3.62)	<0.001	0.0	0.720	0.362
	Other	5	3.22 (1.74–5.94)	<0.001	6.4	0.370	
Study quality	Good	5	2.32 (1.30–4.12)	0.004	0.0	0.468	0.609
	Fair	8	2.82 (1.79–4.46)	<0.001	0.0	0.566	

### Components of MetS in PCOS and non-PCOS individuals

**Figure 3:**
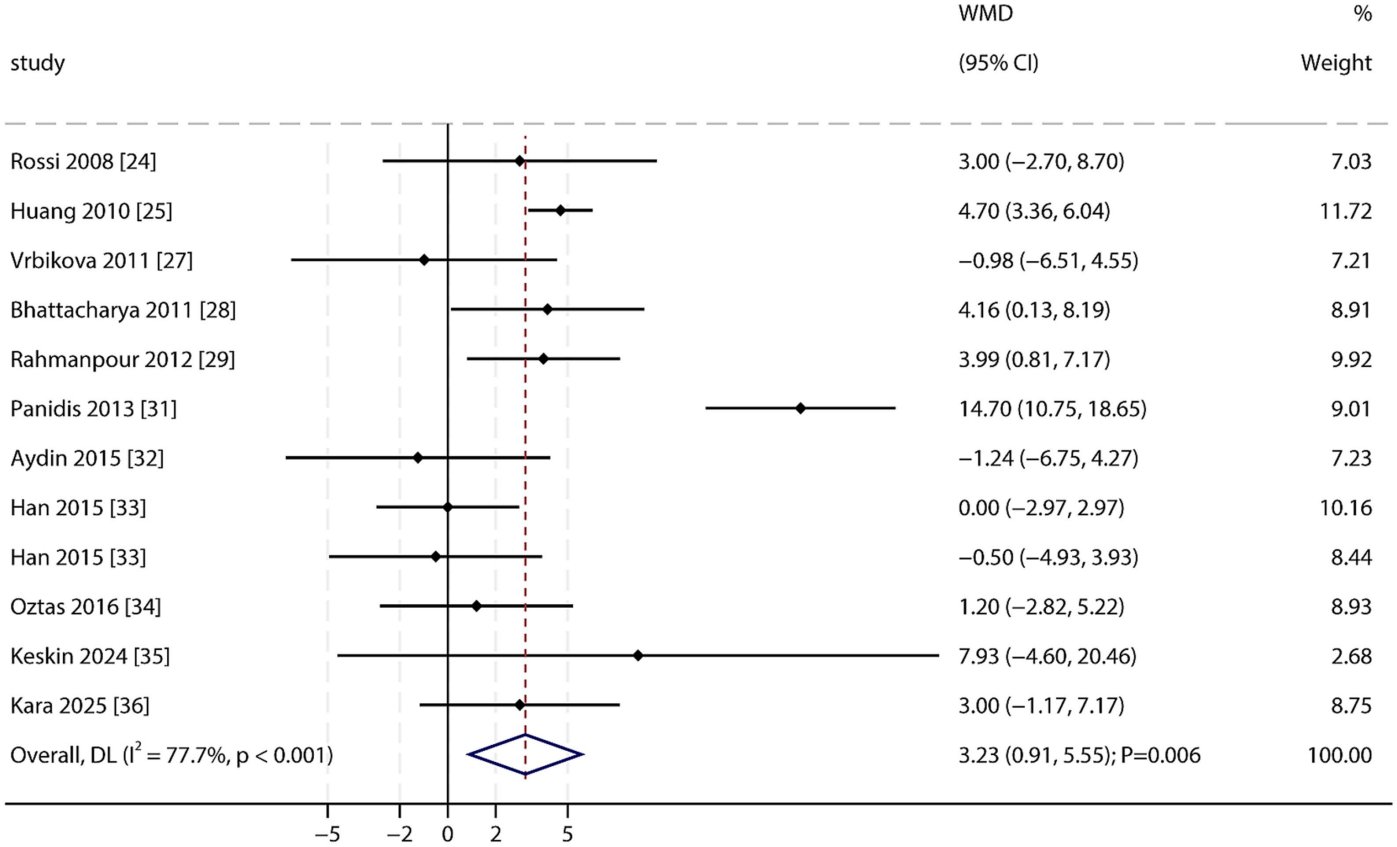
Forest plot of WC between PCOS and non-PCOS. Weights are from random-effects model.

Waist Circumference (WC): A total of 11 studies reported WC data. Meta-analysis demonstrated a significantly higher WC in PCOS patients than in non-PCOS individuals (WMD: 3.23 cm; 95% CI: 0.91–5.55; *p* = 0.006; Figure 3), with significant heterogeneity among studies (*I*^2^ = 77.7%; *p* < 0.001). Sensitivity analysis indicated that the overall result was robust and not driven by any single study (Supplementary File 2). No significant publication bias was detected (Egger’s test *p* = 0.526; Begg’s test *p* = 0.837; Supplementary File 3).

**Figure 4:**
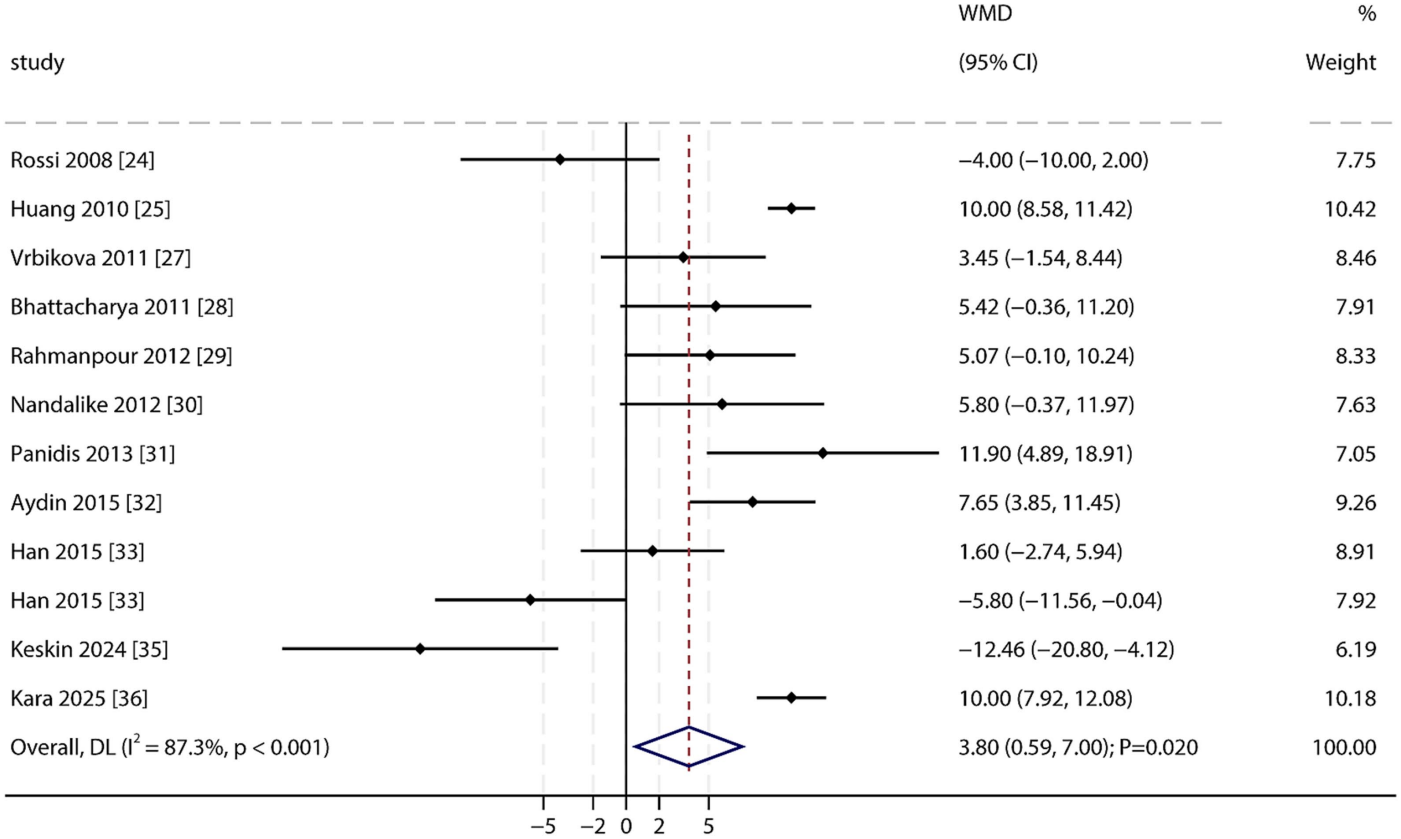
Forest plot of SBP between PCOS and non-PCOS. Weights are from random-effects model.

Systolic Blood Pressure (SBP): A total of 11 studies reported SBP data. The pooled analysis demonstrated a significantly higher SBP in PCOS patients than in non-PCOS individuals (WMD: 3.80 mmHg; 95% CI: 0.59–7.00; *p* = 0.020; Figure 4), with significant heterogeneity among studies (*I*^2^ = 87.3%; *p* < 0.001). Sensitivity analysis indicated that this finding was unstable and sensitive to the removal of individual studies (Supplementary File 2). Furthermore, the results suggested potential publication bias (Egger’s test *p* = 0.003; Begg’s test *p* = 0.150; Supplementary File 3). After applying the trim-and-fill method to adjust for potential bias, the association remained statistically significant.

**Figure 5:**
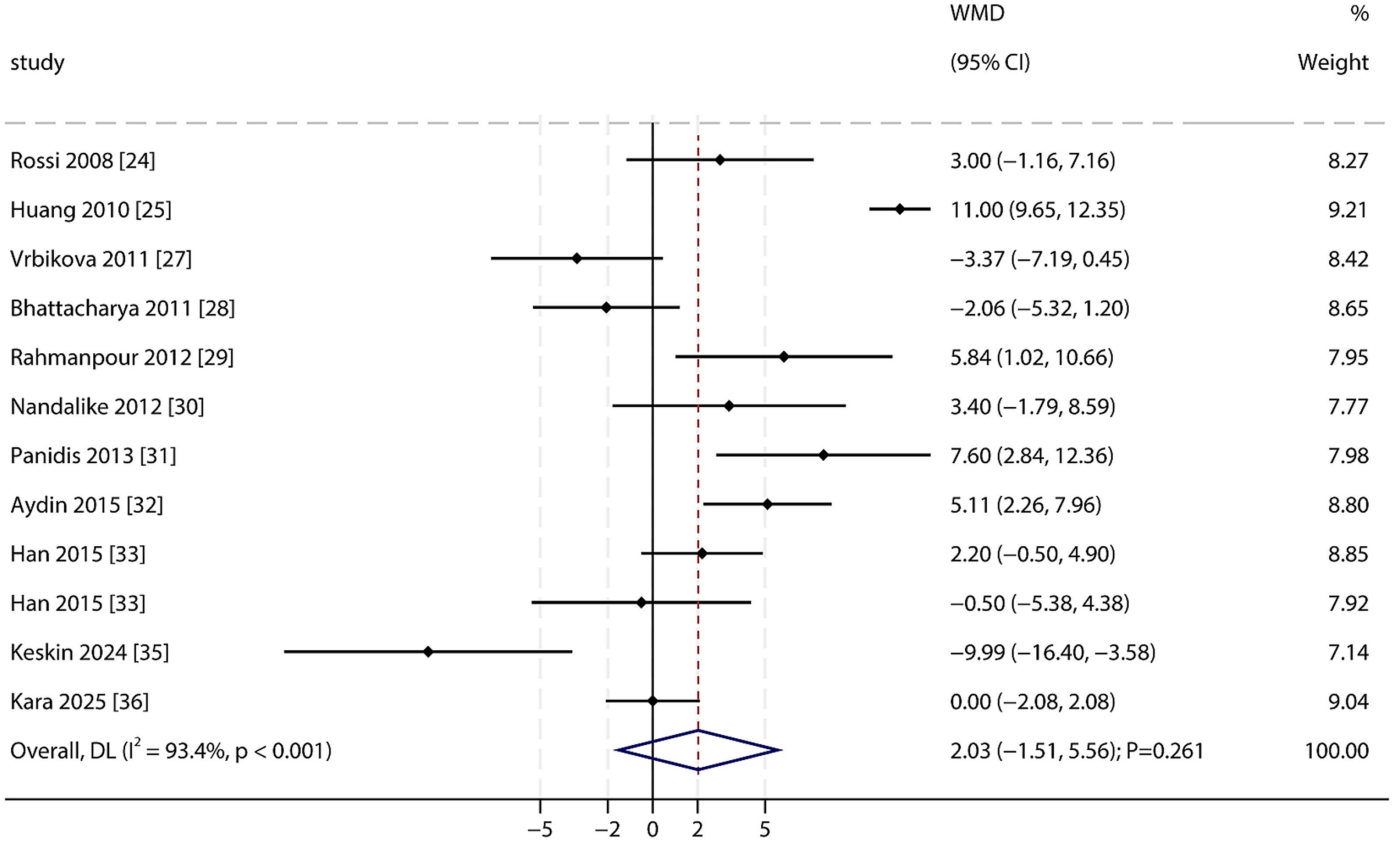
Forest plot of DBP between PCOS and non-PCOS. Weights are from random-effects model.

Diastolic Blood Pressure (DBP): A total of 11 studies reported DBP data. The pooled analysis found no statistically significant difference in DBP between PCOS patients and non-PCOS individuals (WMD: 2.03 mmHg; 95% CI: −1.51 to 5.56; *p* = 0.261; Figure 5), with significant heterogeneity among studies (*I*^2^ = 93.4%; *p* < 0.001). Sensitivity analysis suggested that the overall result was not robust, and DBP might be higher in the PCOS group. No significant publication bias was indicated by Begg’s test (*p* = 0.837), while Egger’s test suggested potential bias (*p* = 0.025; Supplementary File 3). After applying the trim-and-fill method to adjust for potential bias, the results remained non-significant.

**Figure 6:**
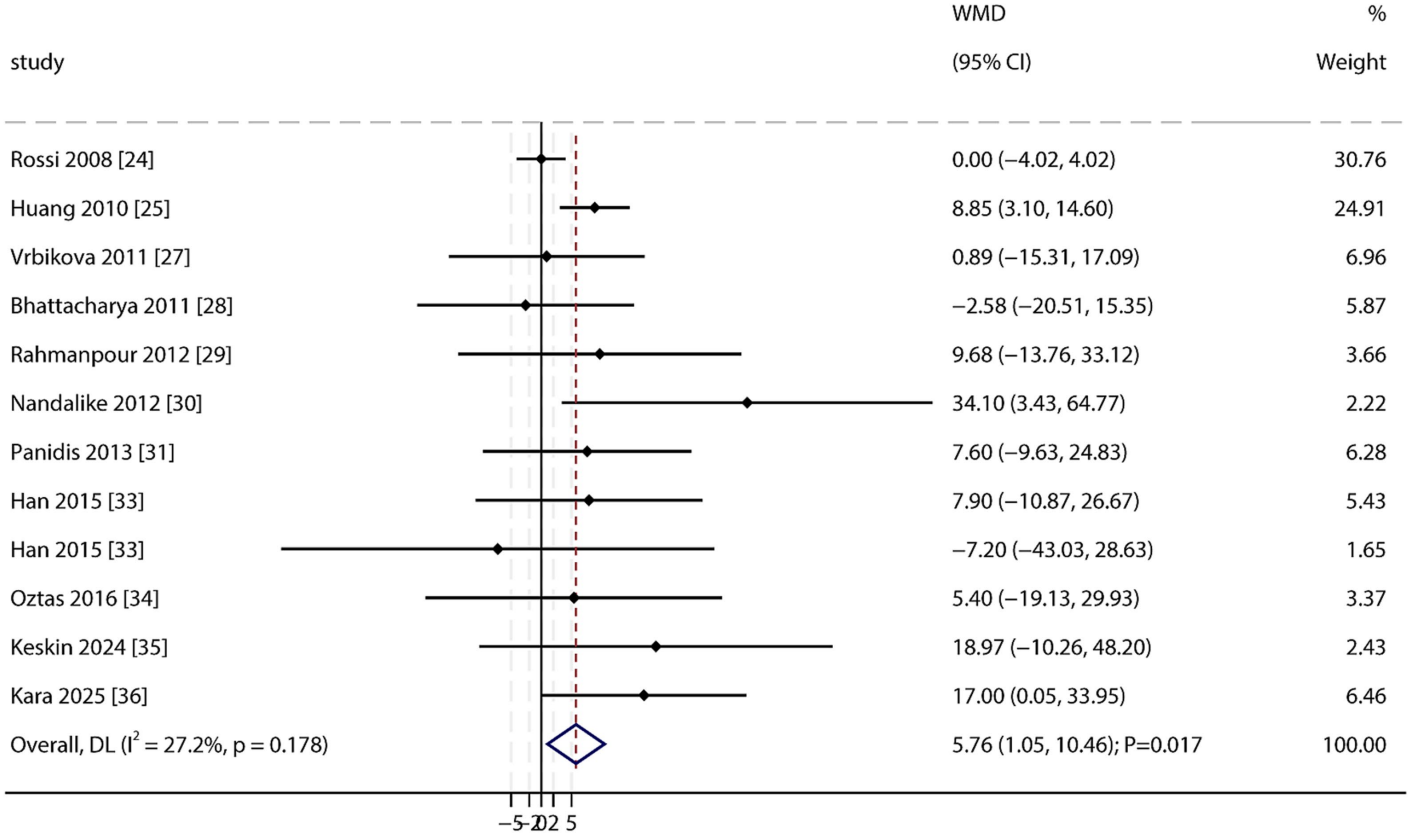
Forest plot of TGs between PCOS and non-PCOS. Weights are from random-effects model.

Triglycerides (TGs): A total of 11 studies reported data on TG levels. A meta-analysis revealed significantly higher TG levels in PCOS patients than in non-PCOS individuals (WMD: 5.76 mg/dL; 95% CI: 1.05–10.46; *p* = 0.017; Figure 6), with no significant heterogeneity among studies (*I*^2^ = 27.2%; *p* = 0.178). However, sensitivity analysis indicated that this finding was not robust. No significant publication bias was detected (Egger’s test *p* = 0.139; Begg’s test *p* = 0.732; Supplementary File 3).

**Figure 7:**
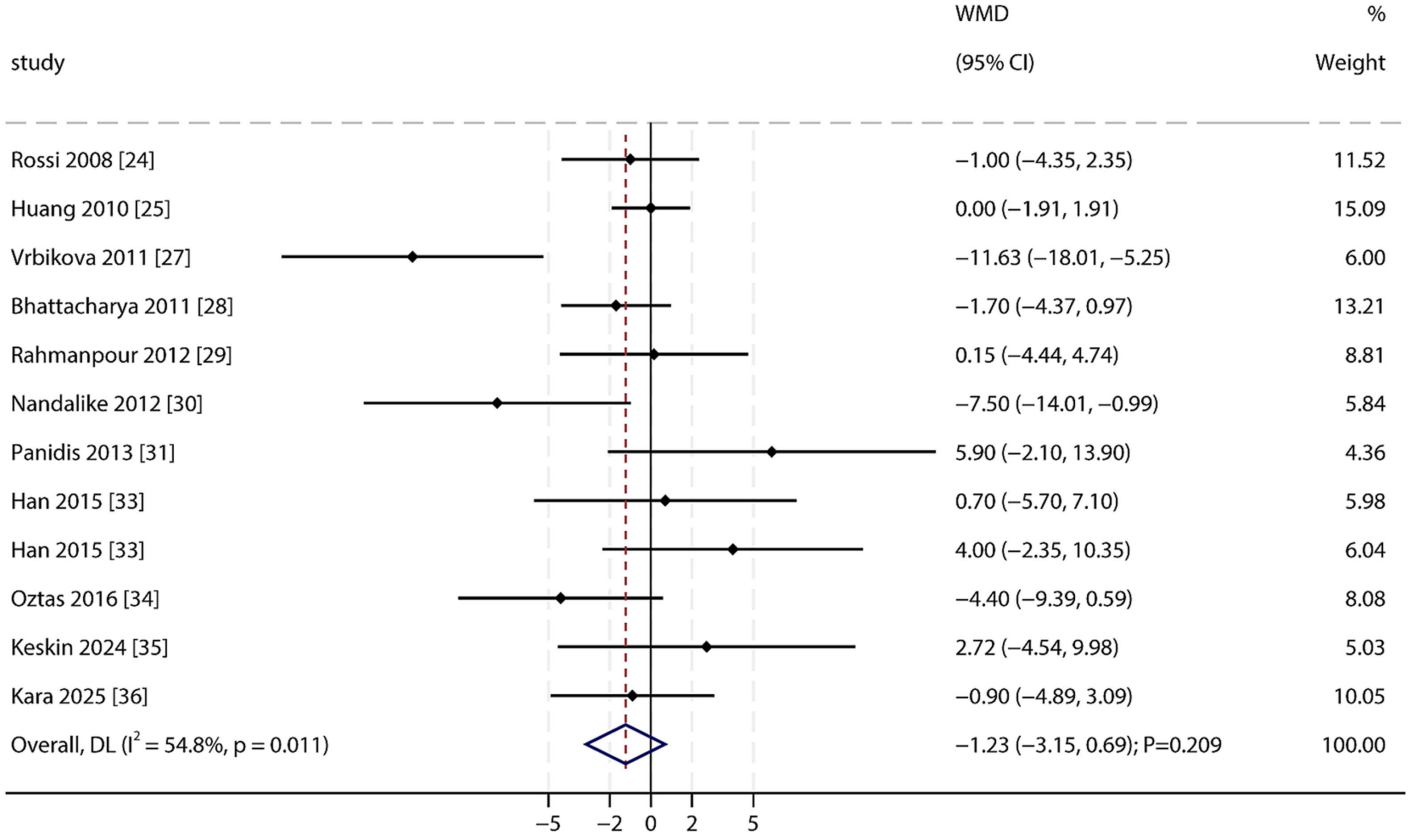
Forest plot of HDL between PCOS and non-PCOS. Weights are from random-effects model.

High-Density Lipoprotein (HDL): A total of 11 studies reported data on HDL levels. The pooled analysis showed no statistically significant difference in HDL levels between PCOS patients and non-PCOS individuals (WMD: −1.23 mg/dL; 95% CI: −3.15 to 0.69; *p* = 0.209; Figure 7), with significant heterogeneity among studies (*I*^2^ = 54.8%; *p* = 0.011). Sensitivity analysis confirmed that this result was robust. No significant publication bias was detected (Egger’s test *p* = 0.698; Begg’s test *p* = 0.537; Supplementary File 3).

**Figure 8:**
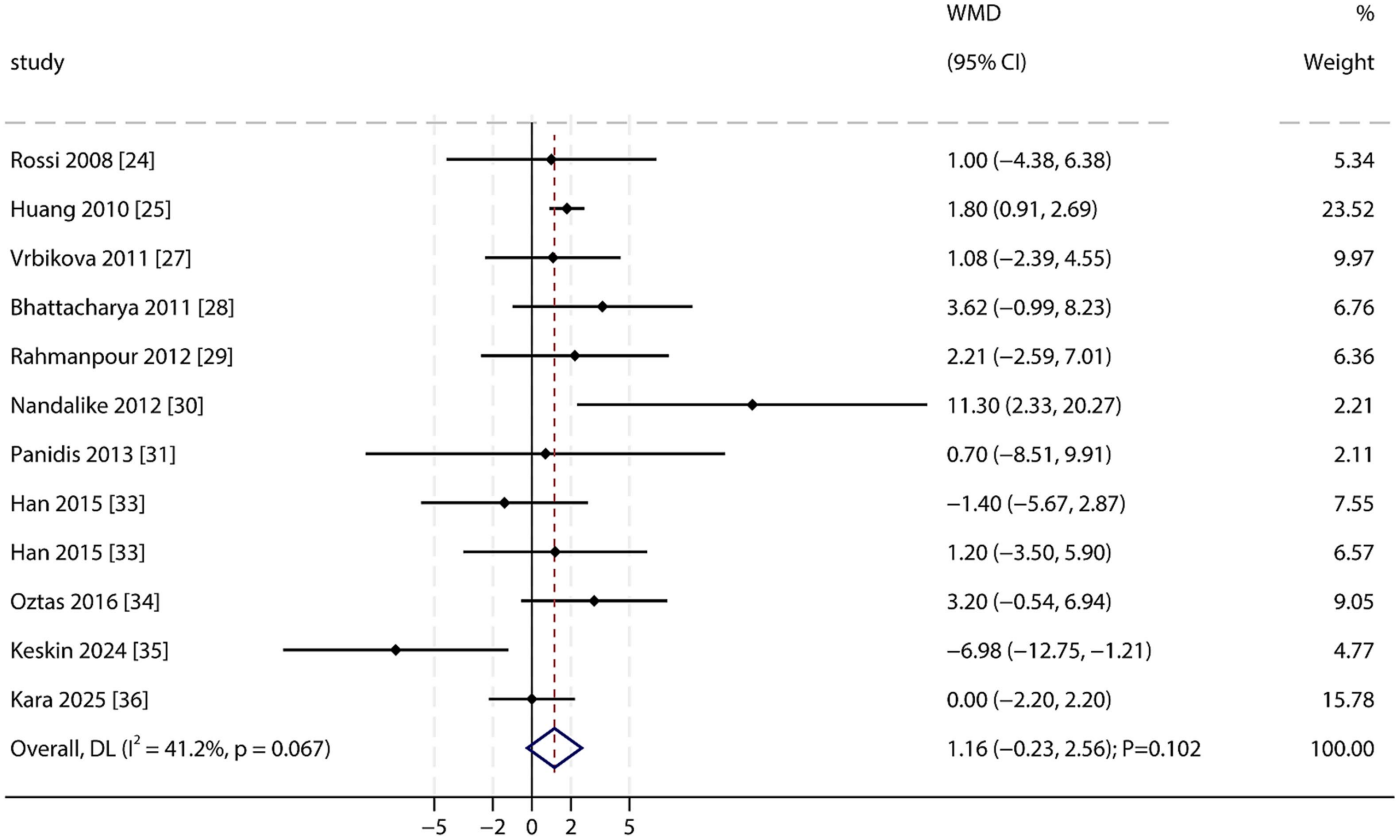
Forest plot of FBG between PCOS and non-PCOS. Weights are from random-effects model.

Fasting Blood Glucose (FBG): A total of 11 studies reported data on FBG levels. A meta-analysis found no statistically significant difference in FBG between PCOS patients and non-PCOS individuals (WMD: 1.16 mg/dL; 95% CI: −0.23 to 2.56; *p* = 0.102; Figure 8). A moderate level of heterogeneity was observed, though it did not reach conventional statistical significance (*I*^2^ = 41.2%; *p* = 0.067). Sensitivity analysis indicated that the overall result was not robust, suggesting that PCOS patients might have higher FBG levels. No significant publication bias was detected (Egger’s test *p* = 0.705; Begg’s test *p* = 1.000; Supplementary File 3).

## Discussion

This systematic review and meta-analysis of 13 observational studies involving 1,789 adolescents quantified the association between PCOS and MetS—along with its individual components—within a unified diagnostic framework specific to adolescents. Our results demonstrate that adolescents with PCOS have a significantly higher risk of developing MetS compared with their non-PCOS peers. Specifically, PCOS patients exhibited significantly higher WC, SBP, and TG levels, whereas no statistically significant differences were observed in DBP, HDL, or FBG. These findings directly address the hypothesis that the PCOS–MetS relationship in adolescents may differ from that in adults and provide a quantitative basis for metabolic risk stratification in clinical practice. Unlike adults with PCOS, who often present with multi-component metabolic abnormalities (11), metabolic disturbances in adolescent PCOS appear to cluster primarily along an “abdominal obesity–blood pressure–lipid” axis, with glucose metabolism remaining largely unaffected at this stage. This pattern may be attributed to the unique interplay of insulin resistance and growth physiology in adolescence. The inherent physiological insulin resistance of puberty, driven by growth hormone surges, creates a background onto which PCOS-associated IR is superimposed. In this context, compensatory hyperinsulinemia may initially suffice to maintain euglycemia, explaining the preserved fasting glucose levels. However, this hyperinsulinemia potently drives hepatic triglyceride synthesis and, through sympathetic activation and renal sodium retention, contributes to early blood pressure elevation. The significant hypertriglyceridemia and elevated SBP observed, alongside marked abdominal obesity—a tissue highly sensitive to insulin’s adipogenic effects—are likely direct manifestations of this pronounced yet compensated hyperinsulinemic state characteristic of early PCOS in youth.

Although the pooled association between PCOS and MetS in our study was statistically significant, the effect size was considerably smaller than that reported in a prior meta-analysis (37). This discrepancy can be attributed to methodological limitations in the earlier studies, including the inclusion of only 12 studies, the potential omission of relevant literature, and the erroneous incorporation of two studies that did not report MetS incidence data. In contrast, our analysis strictly adhered to the following criteria: (1) inclusion of participants aged 10–20 years and (2) application of adolescent-specific MetS diagnostic criteria. This rigorous approach excluded studies with potential contamination from adult data, whereas the earlier meta-analysis might have overestimated the association by including studies with unclear age stratification or those applying adult MetS criteria. The methodological rigor of our study also explains the non-significant difference in FBG observed between PCOS and non-PCOS adolescents. This finding stands in sharp contrast to the elevated FBG commonly observed in adults with PCOS (4). It reinforces the concept of adolescence as a critical window for metabolic intervention—a period during which glucose regulation mechanisms may still be intact, thereby offering an opportunity for early intervention to halt progression toward T2DM.

The distinct pattern of associations between adolescent PCOS and specific MetS components can be explained by the self-reinforcing cycle of “Insulin Resistance—Hyperandrogenism—Abdominal Obesity”: (1) This study found the strongest association with WC. Abdominal obesity, particularly visceral adiposity, is a key driver in this cycle. It acts as an endocrine organ by releasing free fatty acids (FFAs) and pro-inflammatory cytokines, which exacerbate systemic insulin resistance (38). Crucially, the resultant hyperinsulinemia and increased FFA flux to the liver promote hepatic *de novo* lipogenesis and inhibit fatty acid oxidation, establishing a direct pathogenic link to metabolic dysfunction-associated steatotic liver disease (MASLD) (39). MASLD is increasingly recognized in adolescent PCOS and is not merely a hepatic complication but also a contributor to systemic IR and dyslipidemia, thus perpetuating the cycle. In turn, hyperinsulinemia stimulates ovarian androgen production (40). Elevated androgens further promote the accumulation of abdominal fat, creating a vicious cycle (41). This pathophysiological mechanism explains why an elevated WC serves as a primary warning sign for MetS in PCOS patients; (2) insulin resistance can lead to elevated SBP through activation of the renin–angiotensin system (42). Concurrently, it reduces lipoprotein lipase activity, thereby increasing TG synthesis (43). These mechanisms are consistent with our findings of significantly higher SBP and TG levels. The lack of a significant difference in DBP may be attributed to greater aortic elasticity in adolescents, making DBP less sensitive to the effects of insulin resistance compared with adults; and (3) the absence of significantly elevated FBG suggests that pancreatic β-cell function in adolescents remains largely uncompromised (44). At this stage, β-cells can compensate for insulin resistance by increasing insulin secretion, thereby maintaining normoglycemia. However, our sensitivity analysis, which indicated a potential trend toward higher FBG in PCOS patients, underscores the importance of long-term monitoring of glycemic metrics to detect early signs of β-cell function decline.

The role of insulin resistance in adolescent PCOS warrants special consideration. Puberty itself is a state of transient physiological insulin resistance, essential for supporting rapid growth (45). In adolescents with PCOS, this normal physiological process may become exaggerated and pathological. The differentiating factor from adults is the presumed robust functional capacity of pancreatic β-cells in youth (46). Our finding of unchanged fasting glucose, despite evidence of insulin resistance from elevated TGs and WC, supports the model of “compensated insulin resistance” during adolescence. This represents a critical therapeutic window. Lifestyle interventions aimed at improving insulin sensitivity during this period may not only ameliorate current metabolic features, such as dyslipidemia and abdominal adiposity, but also potentially protect β-cell function, delaying or preventing the progression of overt glucose dysregulation seen in adults with PCOS.

Based on our meta-analysis results, we propose a streamlined metabolic screening algorithm for adolescents diagnosed with PCOS. At the time of PCOS diagnosis, we recommend: (1) first-line assessment: WC, SBP, and fasting TGs were measured; (2) risk stratification: the presence of abnormalities in ≥2 of these three core components (using age- and population-specific cutoffs for WC and TGs, and standard pediatric guidelines for elevated SBP) should flag the patient as having a high-risk metabolic phenotype, warranting intensified monitoring and intervention; and (3) comprehensive evaluation: for all adolescents with PCOS, regardless of first-line findings, a full lipid panel and assessment of glucose metabolism (e.g., fasting glucose and/or insulin, HOMA-IR) are prudent to capture individual component risks not fully reflected in the pooled analysis. This algorithm prioritizes efficiency by focusing on the most consistently abnormal metrics while ensuring a complete baseline assessment.

This study has several limitations. First, the inclusion of only two cohort studies precludes a robust establishment of causality between PCOS and MetS. Second, the substantial heterogeneity observed for some components may be attributed to unaccounted confounders, such as BMI and physical activity levels, which were not consistently adjusted for across the primary studies. Furthermore, the results for SBP and TGs were unstable in sensitivity analyses, which may affect the reliability of these specific findings. Third, a notable limitation is the absence of an analysis of androgen levels. While our study focused exclusively on the core components of MetS, the interplay between hyperandrogenism and metabolic dysfunction is central to PCOS. Although all included studies diagnosed PCOS using recognized criteria that incorporate hyperandrogenemia, quantitative androgen data were not consistently reported in a manner that could be pooled. This prevented us from exploring how hormonal status might moderate the risk of MetS or its components, which represents a significant avenue for future research. Fourth, certain studies did not report detailed measurement methods for MetS components, and data on potential confounding conditions, such as MASLD, were not systematically extracted in the primary studies, which limits the ability to fully elucidate the metabolic burden. Finally, as the analysis is based exclusively on the published literature, the potential for publication bias, though tested for, remains an inherent limitation.

## Conclusion

This analysis establishes that adolescents with PCOS face a significantly increased risk of MetS, characterized by a distinct pattern of abdominal obesity, elevated SBP, and hypertriglyceridemia, with preserved glucose metabolism at this stage. This evidence underscores the clinical imperative to routinely incorporate metabolic risk assessment and preemptive intervention into standard care for adolescent PCOS. Central to this effort is addressing abdominal obesity through lifestyle modification, thereby disrupting a key driver of metabolic deterioration. Future studies should prioritize prospective cohort studies to clarify causal pathways and interventional trials to refine effective management strategies.

## Data Availability

The original contributions presented in the study are included in the article/Supplementary material further inquiries can be directed to the corresponding author.

## References

[1] Mousa A Tay CT Teede HJ. Technical Report for the 2023 International Evidence-Based Guideline for the Assessment and Management of Polycystic Ovary Syndrome. Australia: Monash University (2023).

[2] Rosenfield RL Ehrmann DA. The pathogenesis of polycystic ovary syndrome (PCOS): the hypothesis of PCOS as functional ovarian hyperandrogenism revisited. Endocr Rev. (2016) 37:467–520. doi: 10.1210/er.2015-1104, 27459230 PMC5045492

[3] Anderson AD Solorzano CM McCartney CR. Childhood obesity and its impact on the development of adolescent PCOS. Semin Reprod Med. (2014) 32:202–13. doi: 10.1055/s-0034-1371092, 24715515 PMC4103796

[4] Ozegowska K Korman M Szmyt A Pawelczyk L. Heterogeneity of Endocrinologic and metabolic parameters in reproductive age polycystic ovary syndrome (PCOS) women concerning the severity of hyperandrogenemia-a new insight on syndrome pathogenesis. Int J Environ Res Public Health. (2020) 17:9291. doi: 10.3390/ijerph17249291, 33322590 PMC7763600

[5] Alberti KG Eckel RH Grundy SM Zimmet PZ Cleeman JI Donato KA . Harmonizing the metabolic syndrome: a joint interim statement of the international diabetes federation task force on epidemiology and prevention; National Heart, Lung, and Blood Institute; American Heart Association; World Heart Federation; International Atherosclerosis Society; and International Association for the Study of Obesity. Circulation. (2009) 120:1640–5. doi: 10.1161/CIRCULATIONAHA.109.192644 19805654

[6] Lee MK Lee JH Sohn SY Ahn J Hong OK Kim MK . Cumulative exposure to metabolic syndrome in a national population-based cohort of young adults and sex-specific risk for type 2 diabetes. Diabetol Metab Syndr. (2023) 15:78. doi: 10.1186/s13098-023-01030-z, 37095558 PMC10123975

[7] Park JH Jeong I Ko GJ Jeong S Lee H. Development of a predictive model for metabolic syndrome using noninvasive data and its cardiovascular disease risk assessments: multicohort validation study. J Med Internet Res. (2025) 27:e67525. doi: 10.2196/67525, 40315452 PMC12084770

[8] Asghari G Hasheminia M Heidari A Mirmiran P Guity K Shahrzad MK . Adolescent metabolic syndrome and its components associations with incidence of type 2 diabetes in early adulthood: Tehran lipid and glucose study. Diabetol Metab Syndr. (2021) 13:1. doi: 10.1186/s13098-020-00608-1, 33388084 PMC7778813

[9] Luo X Wang Y Wang L Shen Y Ren M. Association between female androgen levels, metabolic syndrome, and cardiovascular disease: an NHANES analysis (2013-2016). Int J Women's Health. (2024) 16:2087–101. doi: 10.2147/IJWH.S475149, 39659294 PMC11628313

[10] Fazleen NE Whittaker M Mamun A. Risk of metabolic syndrome in adolescents with polycystic ovarian syndrome: a systematic review and meta-analysis. Diabetes Metab Syndr. (2018) 12:1083–90. doi: 10.1016/j.dsx.2018.03.014, 29789222

[11] Palma-Leal X Gálvez-Fernández P Camiletti-Moirón D Segura-Jiménez V. Is active commuting associated with metabolic syndrome in adults, adolescents, and children? A systematic review and meta-analysis. Nutr Metab Cardiovasc Dis. (2025) 35:104227. doi: 10.1016/j.numecd.2025.104227, 40914699

[12] Peña AS Witchel SF Boivin J Burgert TS Ee C Hoeger KM . International evidence-based recommendations for polycystic ovary syndrome in adolescents. BMC Med. (2025) 23:151. doi: 10.1186/s12916-025-03901-w, 40069730 PMC11899933

[13] Page MJ McKenzie JE Bossuyt PM Boutron I Hoffmann TC Mulrow CD . The PRISMA 2020 statement: an updated guideline for reporting systematic reviews. BMJ. (2021) 372:n71. doi: 10.1136/bmj.n71 33782057 PMC8005924

[14] Wells G Shea B O’Connell D. The Newcastle-Ottawa Scale (NOS) for Assessing the Quality of Nonrandomised Studies in Meta-Analyses. Ottawa (ON): Ottawa Hospital Research Institute (2009).

[15] DerSimonian R Laird N. Meta-analysis in clinical trials. Control Clin Trials. (1986) 7:177–88. doi: 10.1016/0197-2456(86)90046-2, 3802833

[16] Ades AE Lu G Higgins JP. The interpretation of random-effects meta-analysis in decision models. Med Decis Mak. (2005) 25:646–54. doi: 10.1177/0272989X05282643, 16282215

[17] Deeks JJ Higgins JPT Altman DG. "Analyzing data and undertaking meta-analyses". In: Higgins J Green S, editors. Cochrane Handbook for Systematic Reviews of Interventions 5.0.1. Oxford, UK: The Cochrane Collaboration (2008) chap 9

[18] Higgins JP Thompson SG Deeks JJ Altman DG. Measuring inconsistency in meta-analyses. BMJ. (2003) 327:557–60. doi: 10.1136/bmj.327.7414.557, 12958120 PMC192859

[19] Tobias A. Assessing the influence of a single study in meta-analysis. Stata Tech Bull. (1999) 47:15–7.

[20] Altman DG Bland JM. Interaction revisited: the difference between two estimates. BMJ. (2003) 326:219. doi: 10.1136/bmj.326.7382.219, 12543843 PMC1125071

[21] Egger M Davey Smith G Schneider M Minder C. Bias in meta-analysis detected by a simple, graphical test. BMJ. (1997) 315:629–34. doi: 10.1136/bmj.315.7109.629, 9310563 PMC2127453

[22] Begg CB Mazumdar M. Operating characteristics of a rank correlation test for publication bias. Biometrics. (1994) 50:1088–101. doi: 10.2307/2533446, 7786990

[23] Duval S Tweedie R. Trim and fill: a simple funnel-plot-based method of testing and adjusting for publication bias in meta-analysis. Biometrics. (2000) 56:455–63. doi: 10.1111/j.0006-341x.2000.00455.x, 10877304

[24] Rossi B Sukalich S Droz J Griffin A Cook S Blumkin A . Prevalence of metabolic syndrome and related characteristics in obese adolescents with and without polycystic ovary syndrome. J Clin Endocrinol Metab. (2008) 93:4780–6. doi: 10.1210/jc.2008-1198, 18812482 PMC2626442

[25] Huang J Ni R Chen X Huang L Mo Y Yang D. Metabolic abnormalities in adolescents with polycystic ovary syndrome in South China. Reprod Biol Endocrinol. (2010) 8:142. doi: 10.1186/1477-7827-8-142, 21083920 PMC2994875

[26] Hart R Doherty DA Mori T Huang RC Norman RJ Franks S . Extent of metabolic risk in adolescent girls with features of polycystic ovary syndrome. Fertil Steril. (2011) 95:2347–53. doi: 10.1016/j.fertnstert.2011.03.001 21450287

[27] Vrbíková J Zamrazilová H Sedláčková B Šnajderová M. Metabolic syndrome in adolescents with polycystic ovary syndrome. Gynecol Endocrinol. (2011) 27:820–2. doi: 10.3109/09513590.2010.508851, 20807165

[28] Bhattacharya SM Jha A. Prevalence and risk of metabolic syndrome in adolescent Indian girls with polycystic ovary syndrome using the 2009 'joint interim criteria'. J Obstet Gynaecol Res. (2011) 37:1303–7. doi: 10.1111/j.1447-0756.2010.01516.x, 21535308

[29] Rahmanpour H Jamal L Mousavinasab SN Esmailzadeh A Azarkhish K. Association between polycystic ovarian syndrome, overweight, and metabolic syndrome in adolescents. J Pediatr Adolesc Gynecol. (2012) 25:208–12. doi: 10.1016/j.jpag.2012.02.004 22578482

[30] Nandalike K Agarwal C Strauss T Coupey SM Isasi CR Sin S . Sleep and cardiometabolic function in obese adolescent girls with polycystic ovary syndrome. Sleep Med. (2012) 13:1307–12. doi: 10.1016/j.sleep.2012.07.002, 22921588 PMC3509263

[31] Panidis D Tziomalos K Macut D Kandaraki EA Tsourdi EA Papadakis E . Age- and body mass index-related differences in the prevalence of metabolic syndrome in women with polycystic ovary syndrome. Gynecol Endocrinol. (2013) 29:926–30. doi: 10.3109/09513590.2013.819079, 23885694

[32] Aydin Y Hassa H Burkankulu D Arslantas D Sayiner D Ozerdogan N. What is the risk of metabolic syndrome in adolescents with normal BMI who have polycystic ovary syndrome? J Pediatr Adolesc Gynecol. (2015) 28:271–4. doi: 10.1016/j.jpag.2014.08.011, 26049937

[33] Han Y Kim HS Lee HJ Oh JY Sung YA. Metabolic effects of polycystic ovary syndrome in adolescents. Ann Pediatr Endocrinol Metab. (2015) 20:136–42. doi: 10.6065/apem.2015.20.3.136, 26512349 PMC4623341

[34] Oztas E Ozler S Tokmak A Yilmaz N Celik HT Kazancı FH . Increased levels of serum granzyme-B is associated with insulin resistance and increased cardiovascular risk in adolescent polycystic ovary syndrome patients. Eur J Obstet Gynecol Reprod Biol. (2016) 198:89–93. doi: 10.1016/j.ejogrb.2016.01.009, 26802256

[35] Keskin M Arsoy HA Kara O Sarandol E Koca N Yilmaz Y. Impact of comorbid polycystic ovary syndrome on clinical and laboratory parameters in female adolescents with metabolic dysfunction-associated steatotic liver disease: a cross-sectional study. J Clin Med. (2024) 13:5885. doi: 10.3390/jcm13195885, 39407944 PMC11477162

[36] Kara L Cicek D Sarikaya E Gok E Berber U Siraz UG . Adolescent PCOS and metabolic health: an analysis of fat, muscle, and hormones. Eur J Obstet Gynecol Reprod Biol. (2025) 314:114648. doi: 10.1016/j.ejogrb.2025.114648, 40818213

[37] Fu L Xie N Qu F Zhou J Wang F. The association between polycystic ovary syndrome and metabolic syndrome in adolescents: a systematic review and meta-analysis. Reprod Sci. (2023) 30:28–40. doi: 10.1007/s43032-022-00864-8, 35107824 PMC9810687

[38] Joosten HF van der Kroon PH. Enlargement of epididymal adipocytes in relation to hyperinsulinemia in obese hyperglycemic mice (Ob-Ob). Metabolism. (1974) 23:59–66. doi: 10.1016/0026-0495(74)90104-8, 4808512

[39] Vesković M Šutulović N Hrnčić D Stanojlović O Macut D Mladenović D. The interconnection between hepatic insulin resistance and metabolic dysfunction-associated steatotic liver disease-the transition from an adipocentric to liver-centric approach. Curr Issues Mol Biol. (2023) 45:9084–102. doi: 10.3390/cimb45110570, 37998747 PMC10670061

[40] Geffner ME Kaplan SA Bersch N Golde DW Landaw EM Chang RJ. Persistence of insulin resistance in polycystic ovarian disease after inhibition of ovarian steroid secretion. Fertil Steril. (1986) 45:327–33. doi: 10.1016/S0015-0282(16)49211-3, 3512314

[41] Mårin P Arver S. Androgens and abdominal obesity. Bailliere Clin Endocrinol Metab. (1998) 12:441–51. doi: 10.1016/s0950-351x(98)80191-2 10332565

[42] Preuss HG Echard B Bagchi D Perricone NV. Comparing effects of carbohydrate (CHO) blockers and trivalent chromium on CHO-induced insulin resistance and elevated blood pressure in rats. J Am Coll Nutr. (2013) 32:58–65. doi: 10.1080/07315724.2013.770335, 24015700

[43] Jeppesen J Hollenbeck CB Zhou MY Coulston AM Jones C Chen YD . Relation between insulin resistance, hyperinsulinemia, postheparin plasma lipoprotein lipase activity, and postprandial lipemia. Arterioscler Thromb Vasc Biol. (1995) 15:320–4. doi: 10.1161/01.atv.15.3.320, 7749841

[44] Gungor N Saad R Janosky J Arslanian S. Validation of surrogate estimates of insulin sensitivity and insulin secretion in children and adolescents. J Pediatr. (2004) 144:47–55. doi: 10.1016/j.jpeds.2003.09.045, 14722518

[45] Wells JC Cole TJ. Height, adiposity and hormonal cardiovascular risk markers in childhood: how to partition the associations? Int J Obes. (2014) 38:930–5. doi: 10.1038/ijo.2014.24, 24509503 PMC4088335

[46] Velasquez C Vasquez JS Balcazar N. In vitro effect of fatty acids identified in the plasma of obese adolescents on the function of pancreatic β-cells. Diabetes Metab J. (2017) 41:303–15. doi: 10.4093/dmj.2017.41.4.303, 28868828 PMC5583408

