# Supplementary material for: Association of polycystic ovary syndrome with metabolic syndrome and its components in adolescents: a systematic review and meta-analysis
Source: Front Med (Lausanne). 2026 Mar 26;13:1736558. doi: 10.3389/fmed.2026.1736558 (PMC13062220; doi:10.3389/fmed.2026.1736558)
Supplement: Supplementary file 1 [file Data_Sheet_1.docx]

**PubMed:**

#1 (polycystic ovary syndrome[MeSH Terms]) OR (polycystic ovar* syndrome[Title/Abstract]) OR (PCOS[Title/Abstract]) OR (Stein-Leventhal syndrome[Title/Abstract])

#2 (adolescent[MeSH Terms]) OR (adolescen*[Title/Abstract]) OR (teen*[Title/Abstract]) OR (girl*[Title/Abstract]) OR (young women[Title/Abstract]) OR (pediatric[Title/Abstract]) OR (paediatric[Title/Abstract])

#3 (metabolic syndrome[MeSH Terms]) OR (metabolic syndrome[Title/Abstract]) OR (syndrome X[Title/Abstract]) OR (dysmetabolic syndrome[Title/Abstract])

#4 (insulin resistance[MeSH Terms]) OR (insulin resistan*[Title/Abstract]) OR (hyperinsulinism[Title/Abstract])

#5 (obesity[MeSH Terms]) OR (obes*[Title/Abstract]) OR (overweight[Title/Abstract]) OR (body mass index[Title/Abstract]) OR (BMI[Title/Abstract]) OR (adipos*[Title/Abstract])

#6 (dyslipidemias[MeSH Terms]) OR (dyslipidemia[Title/Abstract]) OR (hyperlipidemia[Title/Abstract]) OR (hypertriglyceridemia[Title/Abstract]) OR (lipid*[Title/Abstract]) OR (cholesterol[Title/Abstract]) OR (triglyceride*[Title/Abstract]) OR (HDL[Title/Abstract]) OR (LDL[Title/Abstract])

#7 (glucose intolerance[MeSH Terms]) OR (glucose intoleran*[Title/Abstract]) OR (impaired fasting glucose[Title/Abstract]) OR (impaired glucose tolerance[Title/Abstract]) OR (hyperglycemia[Title/Abstract])

#8 (hypertension[MeSH Terms]) OR (hypertension[Title/Abstract]) OR (high blood pressure[Title/Abstract]) OR (blood pressure[Title/Abstract])

#9 #3 OR #4 OR #5 OR #6 OR #7 OR #8

#10 #1 AND #2 AND #9

**EmBase:**

#1 exp polycystic ovary syndrome/

#2 (polycystic ovar* syndrome or PCOS or Stein-Leventhal syndrome).ti,ab,kf.

#3 1 or 2

#4 exp adolescent/

#5 (adolescen* or teen* or girl* or "young wom?n" or pediatric or paediatric).ti,ab,kf.

#6 4 or 5

#7 exp metabolic syndrome/

#8 (metabolic syndrome or syndrome X or dysmetabolic syndrome).ti,ab,kf.

#9 exp insulin resistance/

#10 (insulin resistan* or insulin sensitiv* or hyperinsulin?emi*).ti,ab,kf.

#11 exp obesity/

#12 (obes* or overweight or "body mass index" or BMI or adipos*).ti,ab,kf.

#13 exp dyslipidemia/

#14 (dyslipid?emi* or hyperlipid?emi* or hypertriglycerid?emi* or lipid* or cholesterol or triglyceride* or HDL or LDL or "high density lipoprotein" or "low density lipoprotein").ti,ab,kf.

#15 exp glucose intolerance/ or exp impaired glucose tolerance/ or exp hyperglycemia/

#16 ("glucose intoleran*" or "impaired fasting glucose" or "impaired glucose tolerance" or hyperglyc?emi*).ti,ab,kf.

#17 exp hypertension/

#18 (hypertension or "high blood pressure" or "blood pressure").ti,ab,kf.

#19 7 or 8 or 9 or 10 or 11 or 12 or 13 or 14 or 15 or 16 or 17 or 18

#20 3 and 6 and 19

**Cochrane library:**

#1 (polycystic ovar* syndrome OR PCOS) AND (adolescen* OR teen* OR girl* OR pediatric)

#2 (metabolic syndrome OR "insulin resistan*" OR obes* OR dyslipidemia OR hypertensi*)

#3 #1 AND #2

**Web of Science:**

#1 TS=((polycystic ovar* syndrome) OR PCOS OR "Stein-Leventhal syndrome")

#2 TS=((adolescen*) OR teen* OR girl* OR "young women" OR pediatric OR paediatric)

#3 TS=((metabolic syndrome) OR "syndrome X" OR "insulin resistan*" OR obes* OR overweight OR BMI OR dyslipidemia OR cholesterol OR triglyceride* OR "glucose intoleran*" OR hypertension)

#4 #1 AND #2 AND #3
