# Supplementary material for: Association of polycystic ovary syndrome with metabolic syndrome and its components in adolescents: a systematic review and meta-analysis
Source: Front Med (Lausanne). 2026 Mar 26;13:1736558. doi: 10.3389/fmed.2026.1736558 (PMC13062220; doi:10.3389/fmed.2026.1736558)
Supplement: Supplementary file 2 [file Data_Sheet_2.docx]

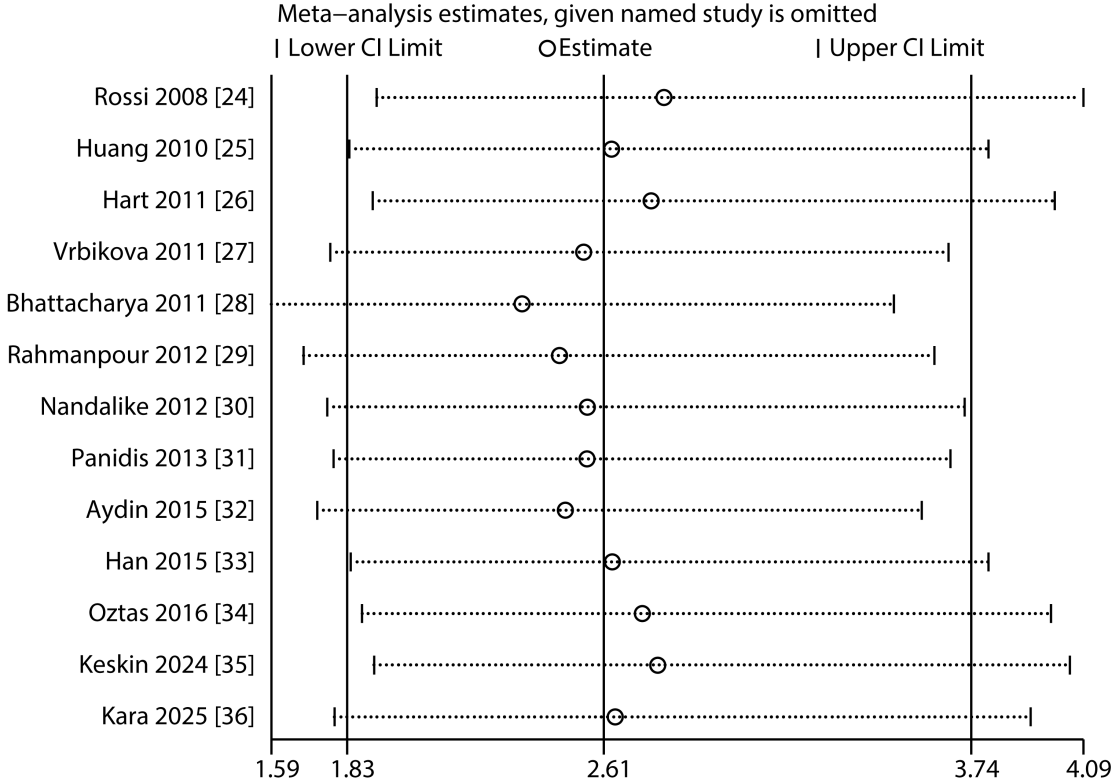


Figure S1. Sensitivity analysis for the association of PCOS with the risk of MetS


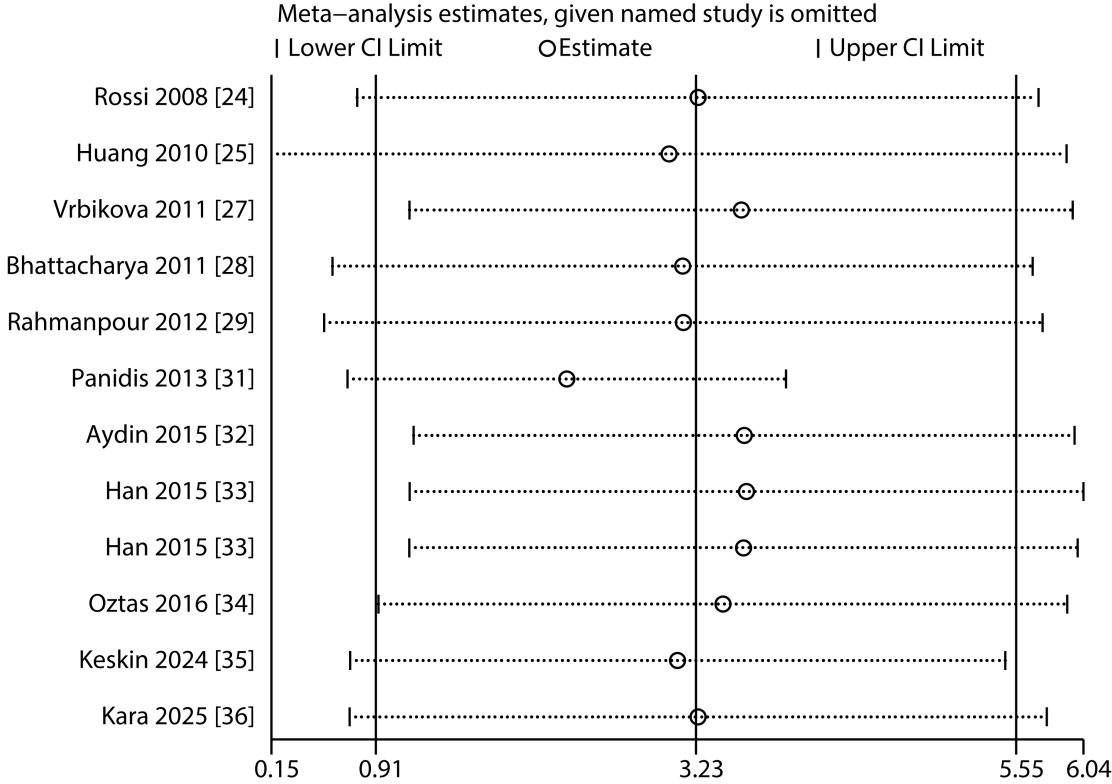


Figure S2. Sensitivity analysis for WC between PCOS and non-PCOS


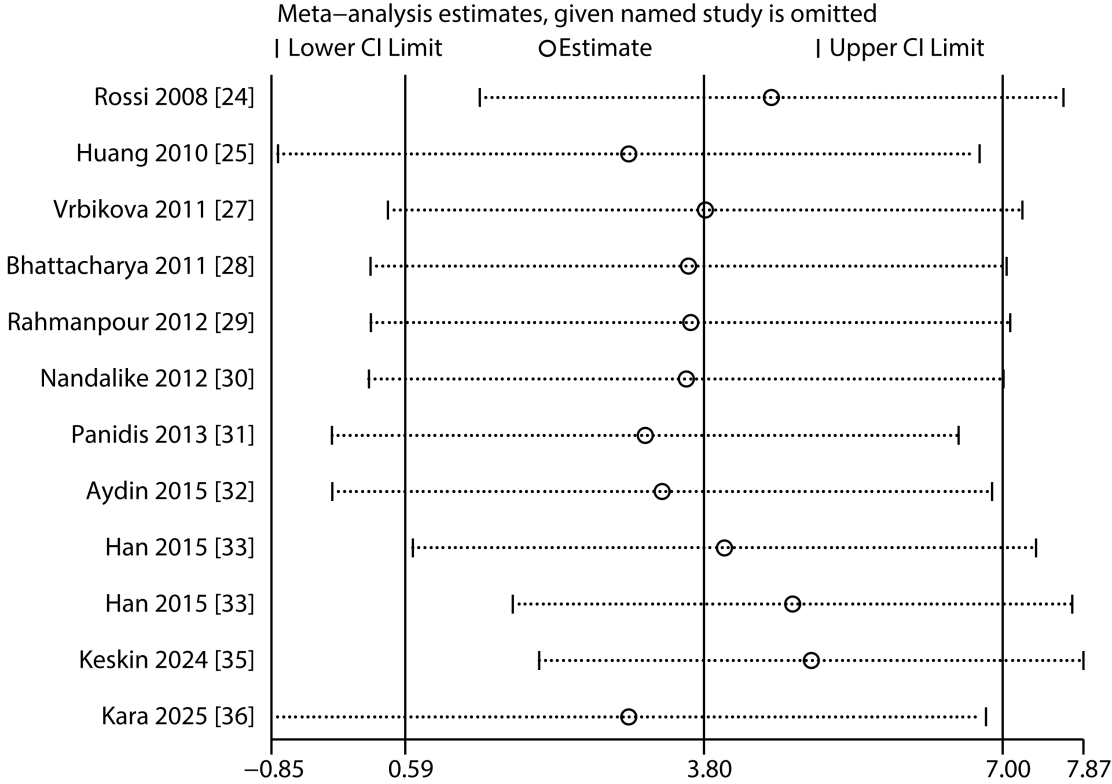


Figure S3. Sensitivity analysis for SBP between PCOS and non-PCOS


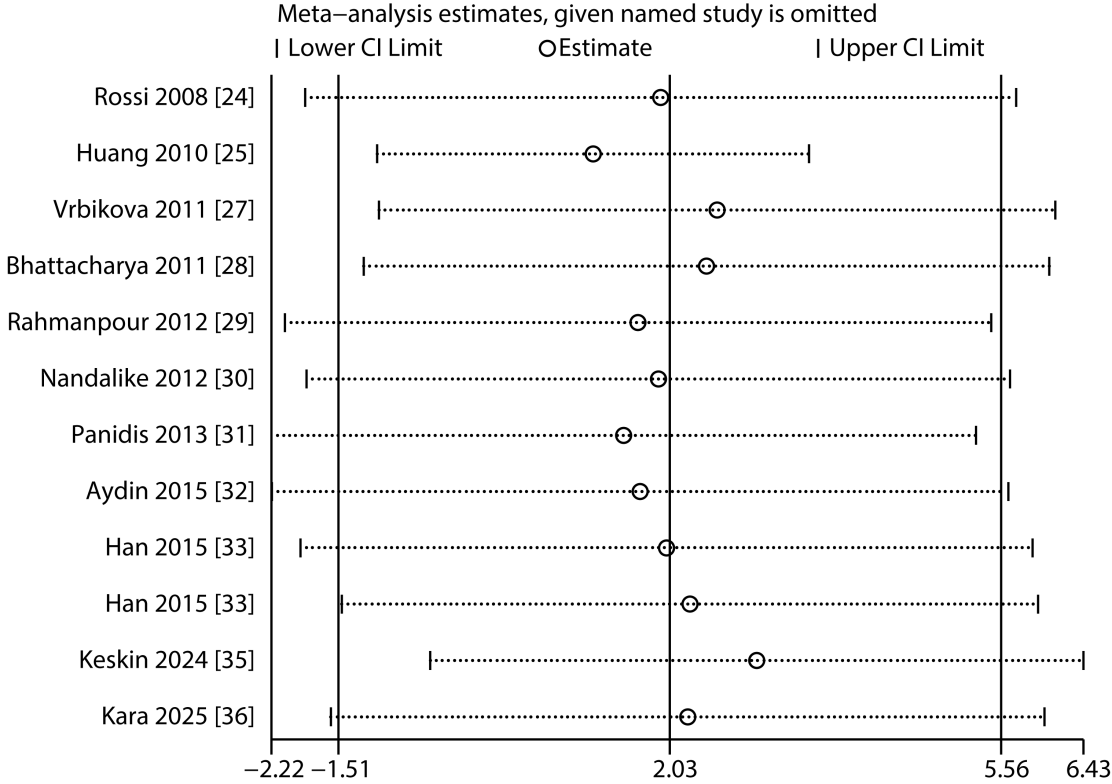


Figure S4. Sensitivity analysis for DBP between PCOS and non-PCOS


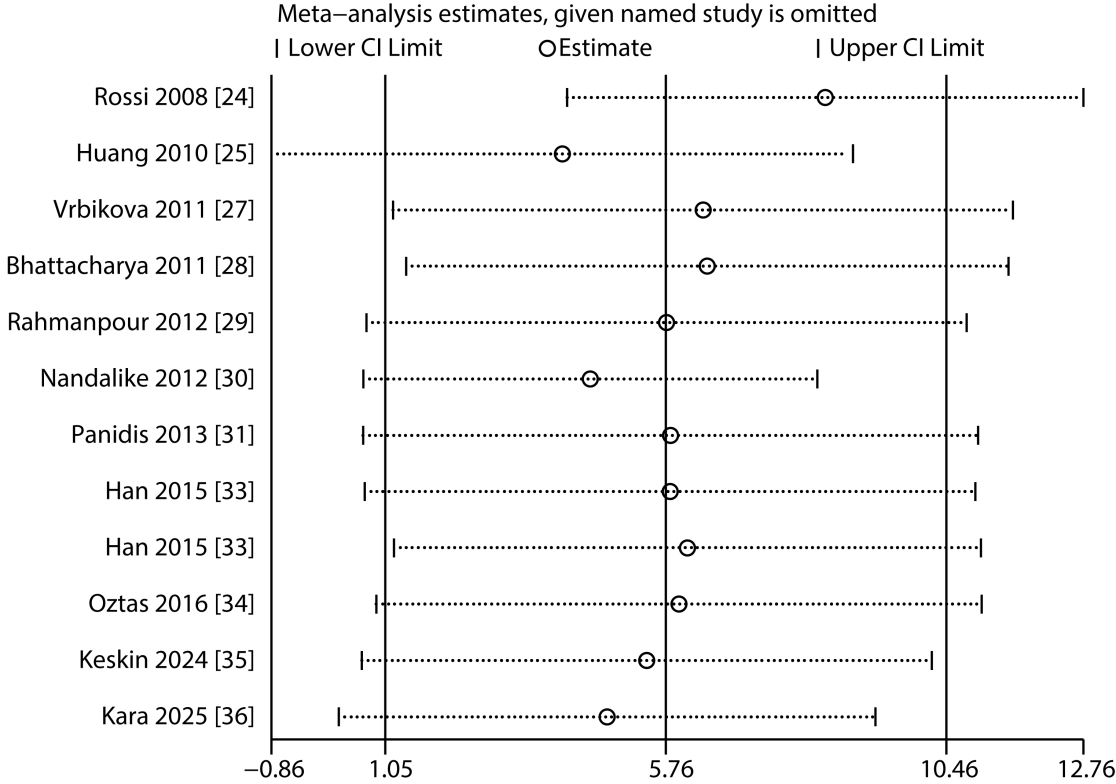


Figure S5. Sensitivity analysis for TG between PCOS and non-PCOS


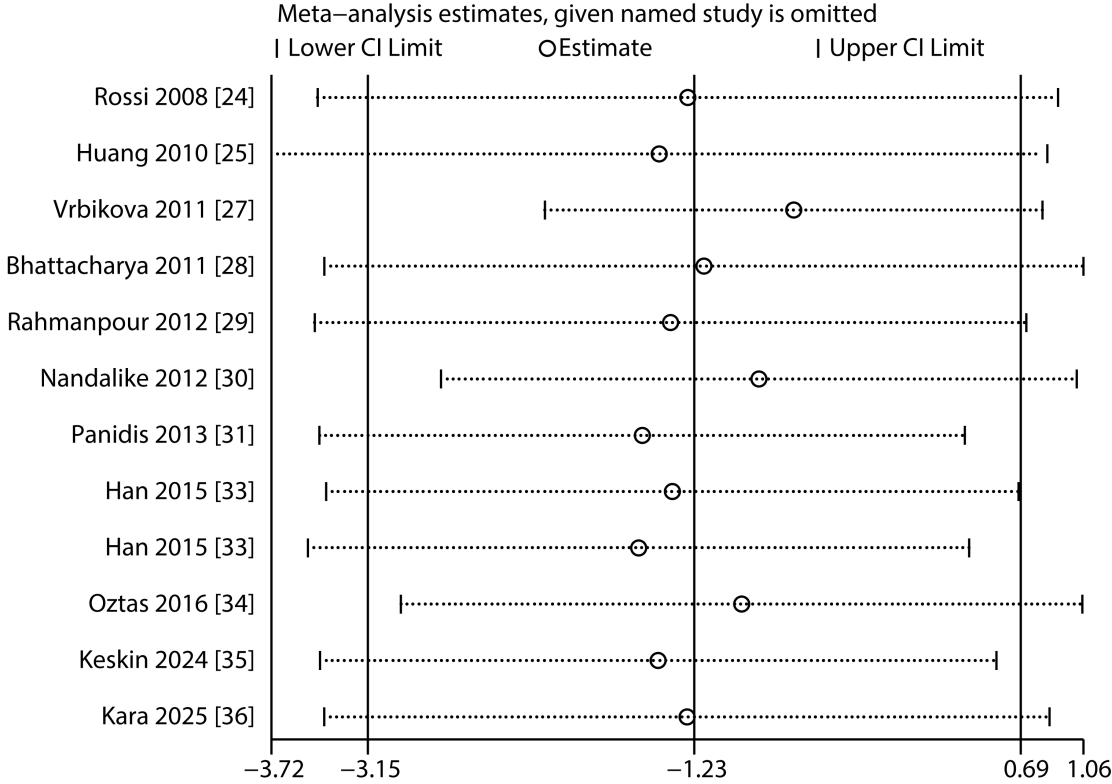


Figure S6. Sensitivity analysis for HDL between PCOS and non-PCOS


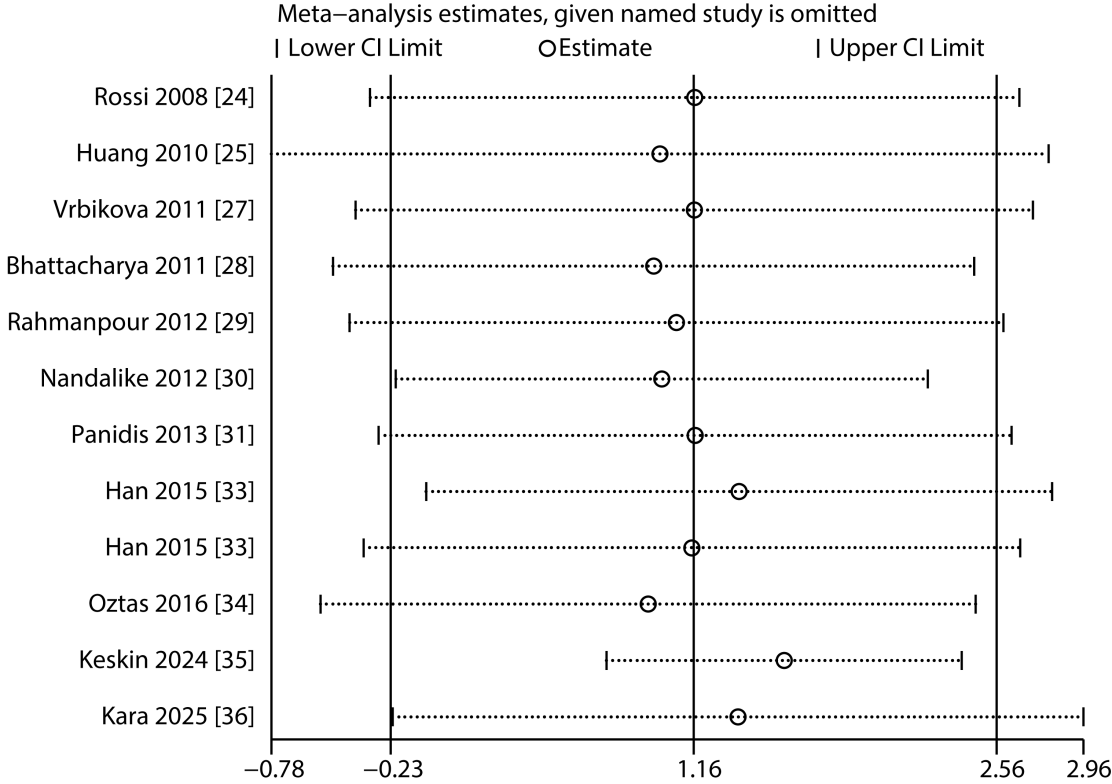


Figure S7. Sensitivity analysis for FBG between PCOS and non-PCOS
