# Supplementary material for: Association of polycystic ovary syndrome with metabolic syndrome and its components in adolescents: a systematic review and meta-analysis
Source: Front Med (Lausanne). 2026 Mar 26;13:1736558. doi: 10.3389/fmed.2026.1736558 (PMC13062220; doi:10.3389/fmed.2026.1736558)
Supplement: Supplementary file 3 [file Data_Sheet_3.docx]

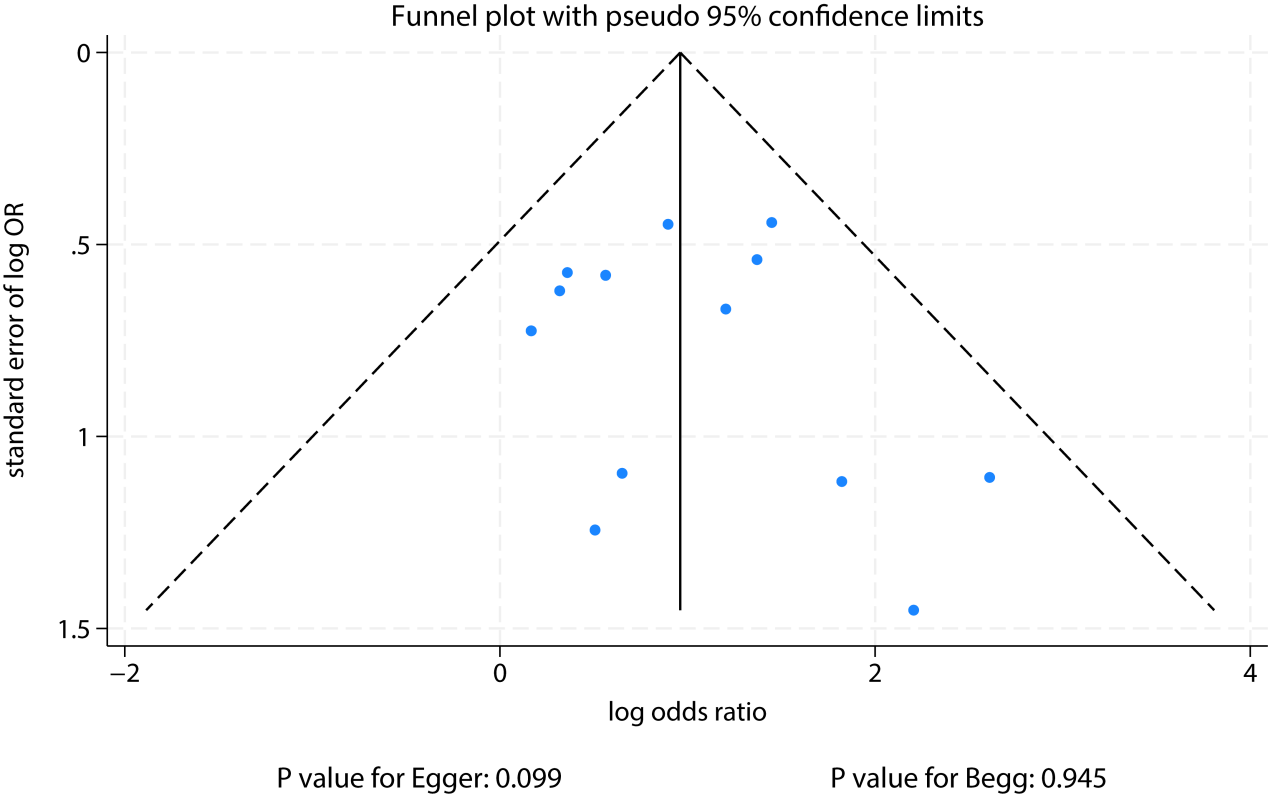


Figure S1. Funnel plot for the association of PCOS with the risk of MetS


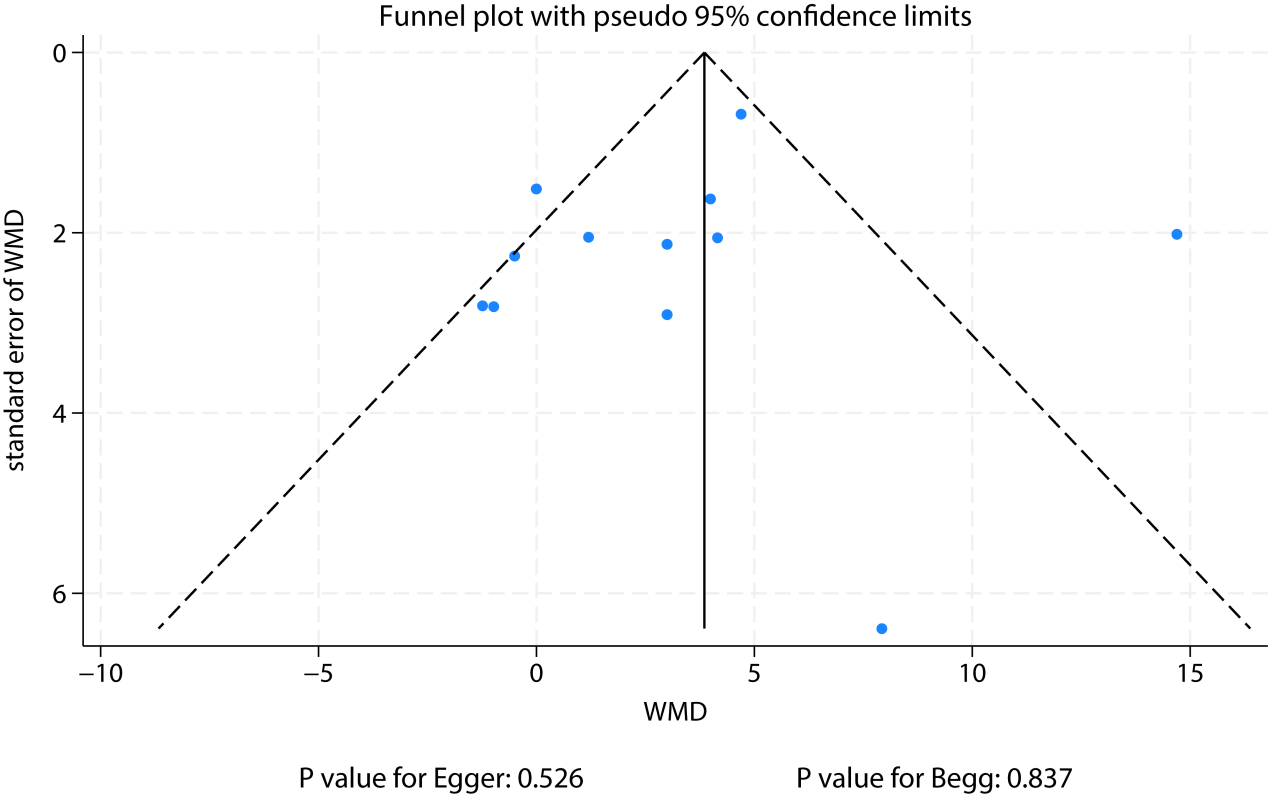


Figure S2. Funnel plot for WC between PCOS and non-PCOS


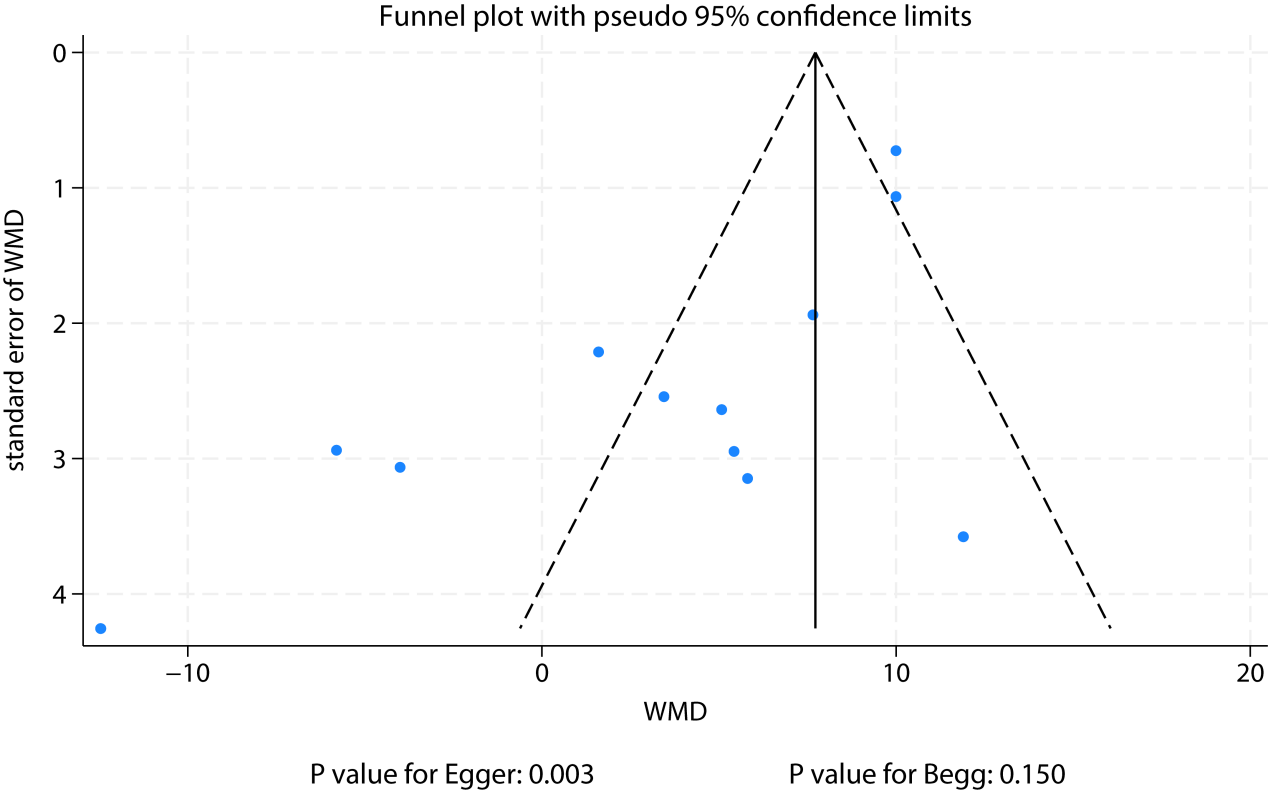


Figure S3. Funnel plot for SBP between PCOS and non-PCOS


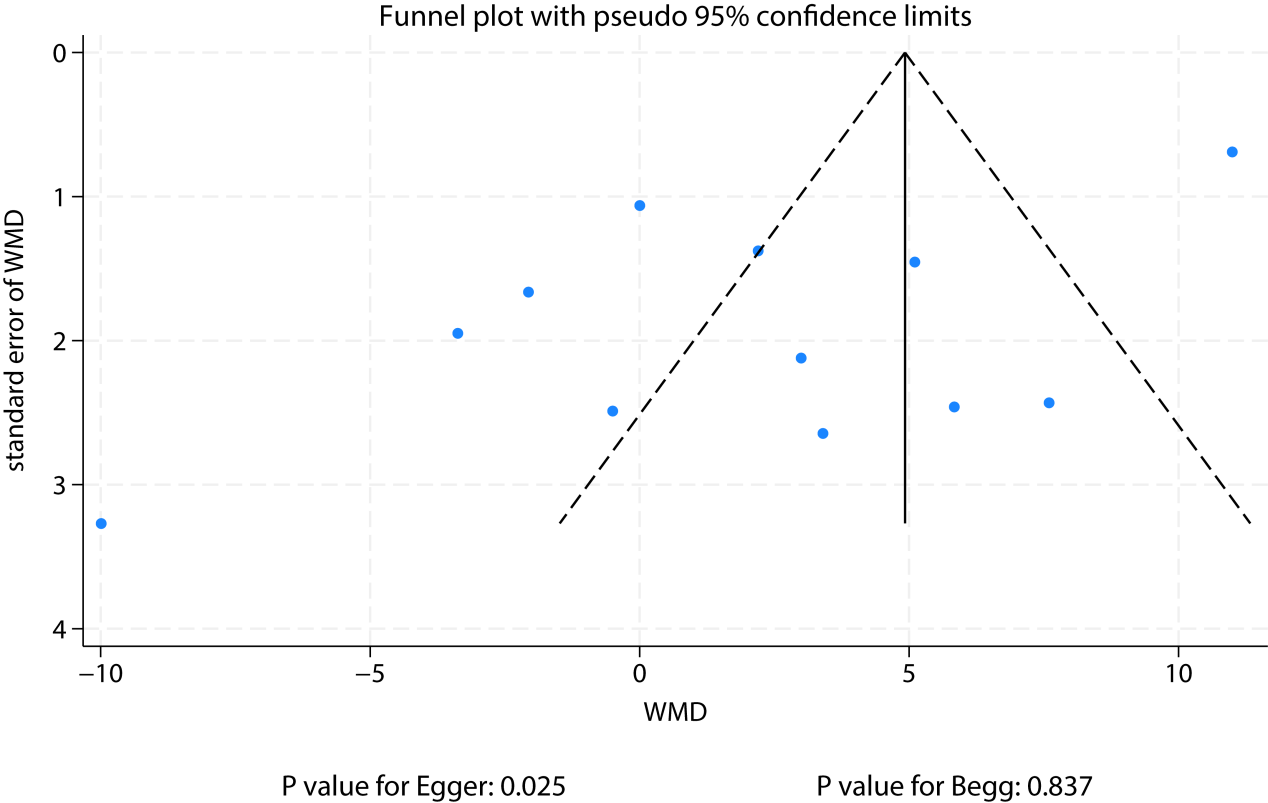


Figure S4. Funnel plot for DBP between PCOS and non-PCOS


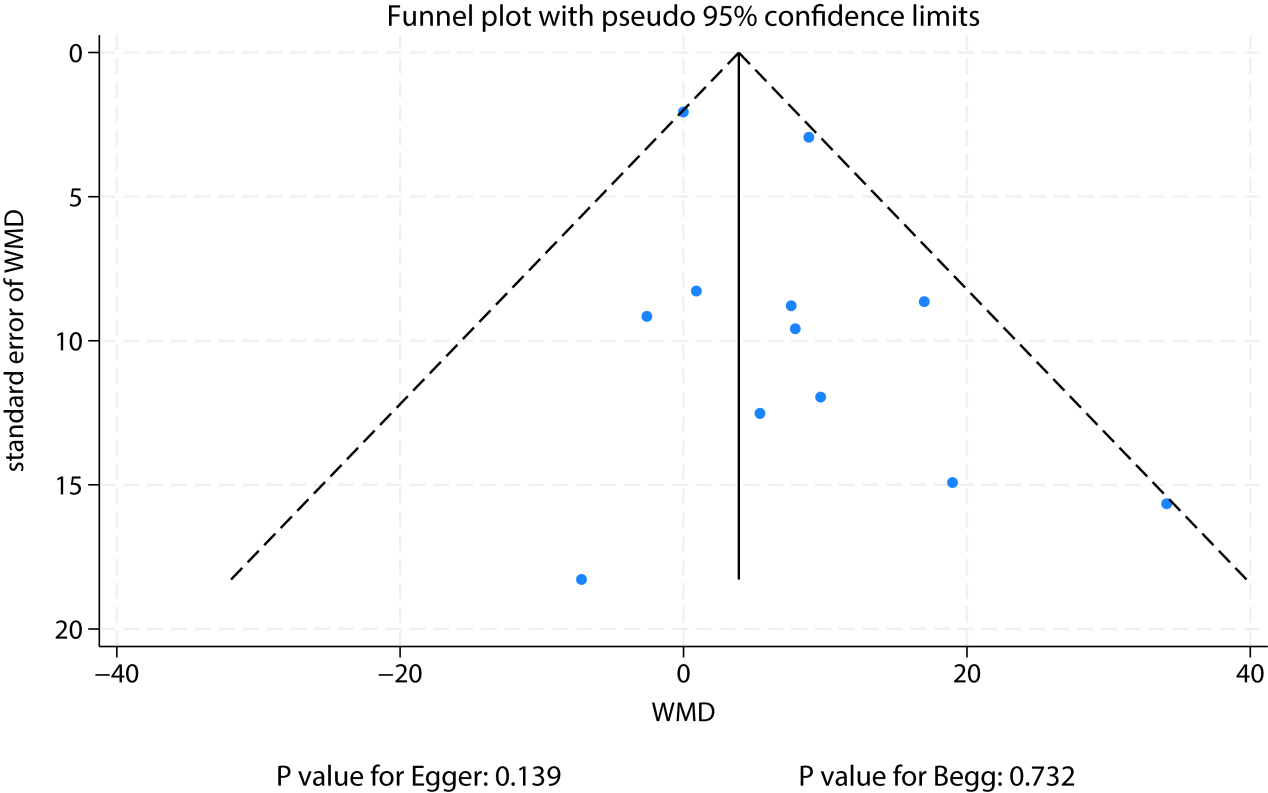


Figure S5. Funnel plot for TG between PCOS and non-PCOS


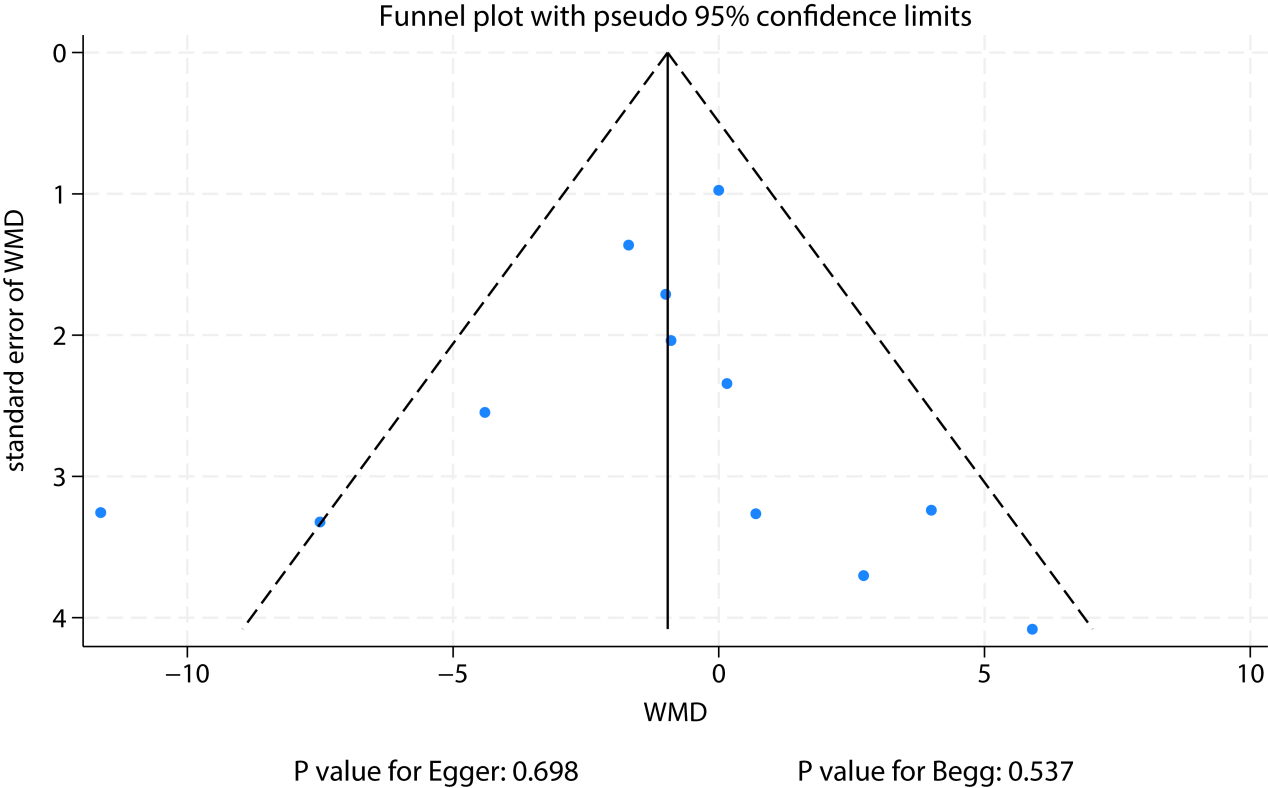


Figure S6. Funnel plot for HDL between PCOS and non-PCOS


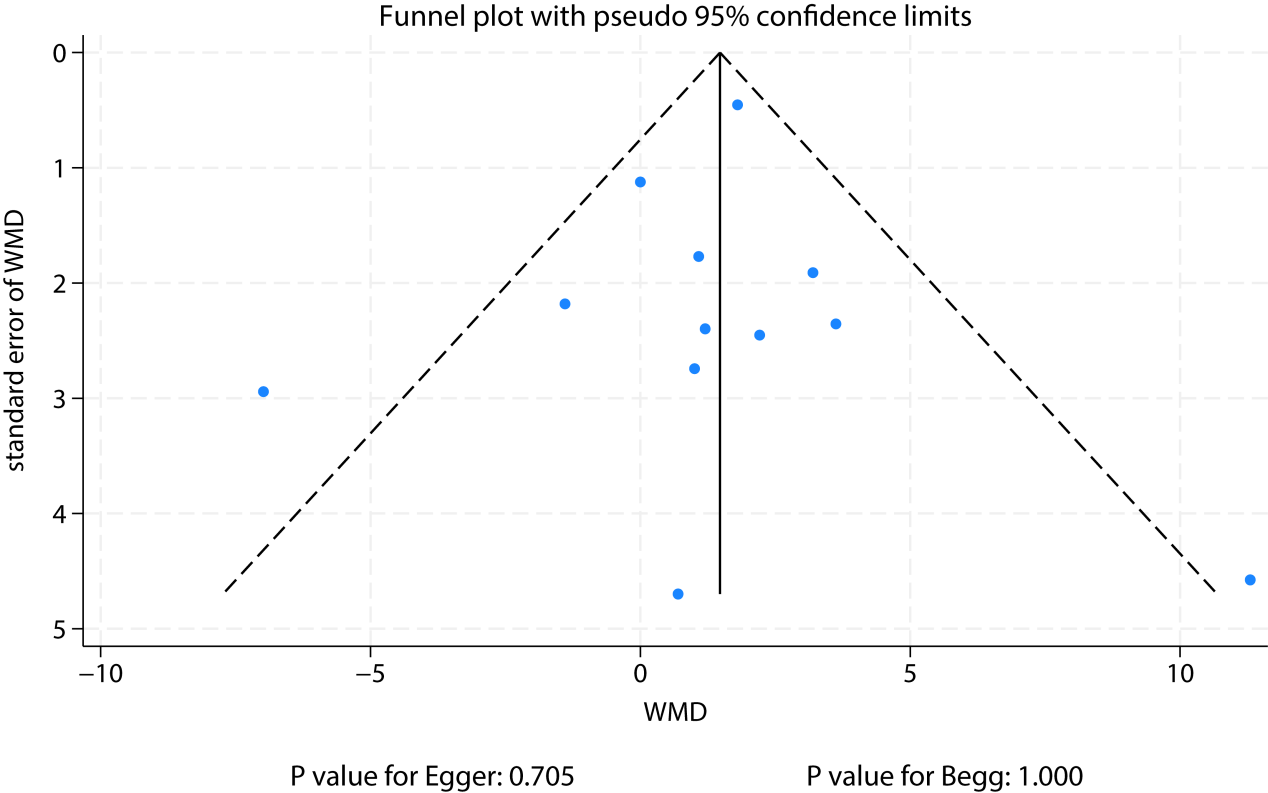


Figure S7. Funnel plot for FBG between PCOS and non-PCOS
